# Electrochromism in Isoreticular Metal–Organic Framework Thin Films with Record High Coloration Efficiency

**DOI:** 10.1021/acsnano.3c06621

**Published:** 2023-10-18

**Authors:** Amol Kumar, Jingguo Li, A. Ken Inge, Sascha Ott

**Affiliations:** † Department of Chemistry - Ångström Laboratory, Uppsala University, Box 523, 75120 Uppsala, Sweden; ‡ Department of Materials and Environmental Chemistry, Stockholm University, 106 91 Stockholm, Sweden

**Keywords:** metal−organic framework, electrochromic materials, coloration efficiency, cyclic voltammetry, electron transport, spectroelectrochemistry

## Abstract

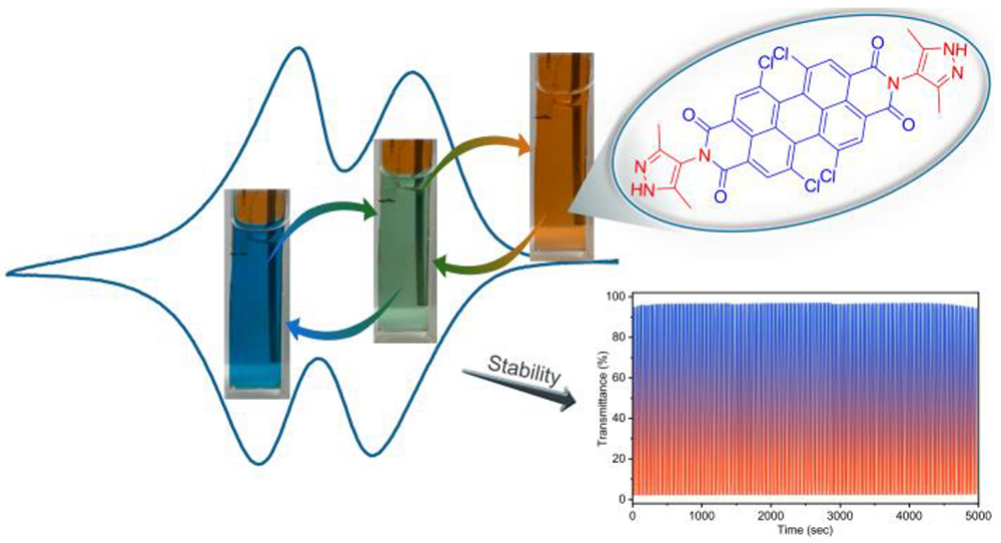

The power of isoreticular chemistry has been widely exploited to engineer metal–organic frameworks (MOFs) with fascinating molecular sieving and storage properties but is underexplored for designing MOFs with tunable optoelectronic properties. Herein, three dipyrazole-terminated XDIs (X = PM (pyromellitic), N (naphthalene), or P (perylene); DI = diimide) with different lengths and electronic properties are prepared and employed as linkers for the construction of an isoreticular series of Zn-XDI MOFs with distinct electrochromism. The MOFs are grown on fluorine-doped tin oxide (FTO) as high-quality crystalline thin films and characterized by X-ray diffraction (XRD) and scanning electron microscopy (SEM). Due to the constituting electronically isolated XDI linkers, each member of the isoreticular thin film series exhibits two reversible one-electron redox events, each at a distinct electrochemical potential. The orientation of the MOFs as thin films as well as their isoreticular nature results in identical cation-coupled electron hopping transport rates in all three materials, as demonstrated by comparable apparent electron diffusion coefficients, *D*_e_^app^. Upon electrochemical reduction to either the [XDI]^•–^ or [XDI]^2–^ state, each MOF undergoes characteristic changes in its optical properties as a function of linker length and redox state of the linker. Operando spectroelectrochemistry measurements reveal that Zn-PDI@FTO (PDI = perylene diimide) thin films exhibit a record high coloration efficiency of 941 cm^2^ C^–1^ at 746 nm, which is attributed to the maximized Faradaic transformations at each electronically isolated PDI unit. The electrochromic response of the thin film is retained to more than 99% over 100 reduction–oxidation cycles, demonstrating the applicability of the presented materials.

## Introduction

Metal–organic frameworks (MOFs) are a class of highly crystalline, porous materials that are composed of metal-based secondary binding units (SBUs) and polydentate organic linkers.^1,2^ Due to their modular nature and tunability, MOFs are extensively investigated for a number of applications ranging from gas separation,^3,4^ catalysis,^5,6^ and enzyme encapsulation^7^ to energy storage technologies^8^ and electronic devices.^9−13^ Designing extended frameworks with desired structures and properties is a central goal in the field and is often achieved by using the principles of reticular chemistry where either the linker or the node of a known MOF is altered to generate analogous MOF topologies with desired porosity and functionality.^14^ The geometric and chemical composition of the SBUs and organic linkers can often be used to predict framework topology,^15^ especially if a base structure is known and can be extended upon.^16,17^

Prolonged linker length with identical SBUs gives rise to identical framework topologies with the only difference being the linker length and thus the dimensions of pore channels and window apertures. In one of the most impressive reports, a phenylene-based linker in MOF-74 has been extended to up to 11 aromatic units, leading to increased pore apertures from 14 to 98 Å.^18^ Such expansions have been used extensively for gas sorption and separation applications^4,19^ but to a much lesser extent for the construction of materials with differing optical properties. Tailored optical properties would be intrinsic to an isoreticular series of MOFs in case the linkers of different lengths are composed of conjugated π-systems.^20−22^ In such systems, similar microscopic charge propagation mechanisms would be expected, presenting the opportunity for fundamental insights into these processes. Moreover, when grown on transparent conducting substrates, the MOFs will engage in electrochromism, the extent of which again will depend on the extent of the π-systems of the linkers in the isoreticular series.

In general, electrochromic behavior is enabled by a reversible change in the optical properties by means of a redox reaction under an applied voltage. Among various electrochromic materials, inorganic metal oxides are the most widely used,^23^ largely due to their compositional stability. Disadvantageous is that these materials are somewhat limited in terms of color tuning versatility, coloration efficiency, and color saturation. In contrast, many of the conducting polymers and small organic molecules provide striking color variations but often suffer from lower long-term stability.^24,25^ Additionally, both metal oxides and conducting polymers are limited by a sluggish switching speed. Although many of these materials are promising and some are commercially available, energy-efficient materials with fast response times are still in demand. Applications of such materials could, for example, be smart windows (e.g., adjustable darkening windows in commercial airlines), antiglazing mirrors in automobiles, and military camouflage.^26,27^

Over recent years, several groups have successfully demonstrated MOF-based electrochromic materials.^28,29^ In 2013, Dincă’s group reported a series of naphthalene diimide (NDI)-based MOFs and achieved multicolor electrochromic performance as a function of differing linker substituents.^30^ A coloration efficiency of 297 cm^2^ C^–1^ at 471 nm was reported for the Zn-NDI film (the second case in our isoreticular series, vide infra) using 0.1 M [(nBu)_4_N]PF_6_ as electrolyte. Rougier’s group constructed a double-sided electrochromic device using a combination of HKUST-1 and ZnMOF-74,^31^ while Farha and Hupp’s groups used pyrene as chromophore and synthesized a free-standing thin film of NU-901 MOF with reversible electrochromic switching ability.^32^ While these examples are important, an isoreticular series of MOFs in which variably sized linkers are composed of conjugated π-systems that impart different colors to the MOF and engage in electrochromism has not been constructed and investigated. Such a series would provide for a detailed investigation and analysis of the electrochemical response and coloration efficiency at the fundamental and applied levels.

**Figure 1:**
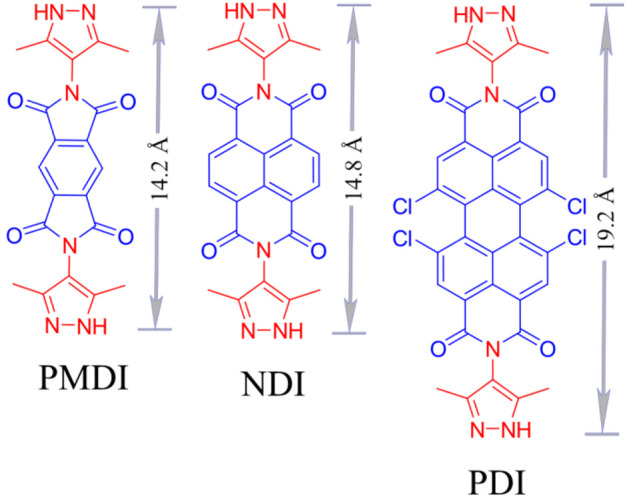
Pyromellitic diimide (PMDI), naphthalene diimide (NDI), and perylene diimide (PDI) bispyrazoles that were used as linkers to construct the isoreticular Zn-XDI (X = PM, N, P) MOF series.

In this contribution, we report a series of isoreticular electrochromic MOFs with Zn^2+^-based SBUs that are interconnected by three different redox-active diimide-containing linkers, i.e., pyromellitic diimide (PMDI), naphthalene diimide (NDI), and perylene diimide (PDI) (Figure 1). Owing to their differing π-systems, these diimides absorb different parts of the visible spectrum. For spectroelectrochemical investigations and future applications as advanced electrochromic materials, thin films of the Zn-XDI (X = PM (pyromellitic), N (naphthalene), or P (perylene); DI = diimide) MOFs were grown on transparent conductive fluorine-doped tin oxide (FTO) substrates. The resulting Zn-XDI@FTO displays reversible Faradaic electrochromic response due to the electronically isolated XDI unit while maintaining structural integrity;^33^ in particular, Zn-PDI retains 98% of the optical contrast even after 150 oxidation/reduction cycles. We demonstrate that the best MOF of the series, Zn-PDI, has a record high coloration efficiency of 941 cm^2^ C^–1^ at 746 nm, which is to the best of our knowledge the highest ever reported for a MOF.

## Results and Discussion

### Linker Design, Isoreticular MOF Synthesis, and Basic Characterization

Owing to the electron deficiency of imides, diimide derivatives of aromatic compounds display strong electron affinity and high extinction coefficients, both of which are essential parameters for electrochromic materials.^34^ When incorporated as linkers in MOFs, the differently sized aromatics offer the opportunity to construct an isoreticular MOF series that differs in the electronic properties of the linkers while maintaining key structural and topological parameters. Thus, three bis-pyrazole-terminated diimides of different lengths and with characteristic electronic properties were designed and synthesized. More specifically, PMDI, NDI, and PDI were employed as core structures of the linkers (Figure 1; see General Methods and SI for synthetic details and ^1^H NMR data in Figures S1–S3). The PDI linker carries additional chloride substituents which can be expected to modulate the electronic properties of the core but are, however, crucial for linker solubility and successful MOF preparation. The number of π-electrons in the diimide cores of the three linkers increases from 18 to 22 and 32 when going from PMDI to NDI and further to PDI, which effectively modulates the HOMO–LUMO energy levels of the linkers (absorption data of free linkers shown in Figure S4).

**Figure 2:**
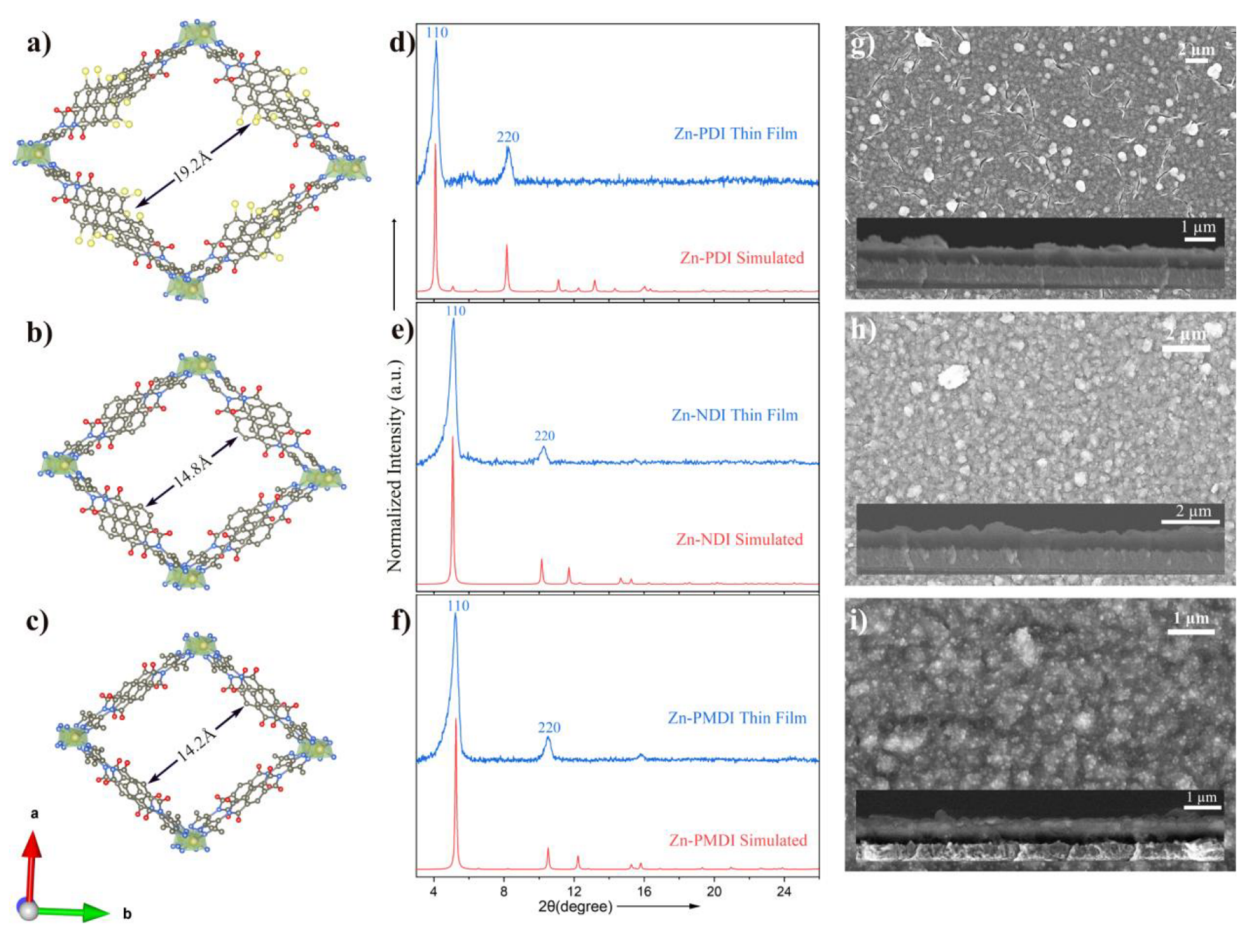
Simulated structure modes of Zn-XDI (X = PM, N, P) with different dimensions of window apertures (a–c); simulated (red) and thin film X-ray diffraction patterns (blue, theta to theta measurements) of the isoreticular series (d–f) and top view and cross-section SEM images of FTO-grown Zn-XDI thin films; (a, d, g) Zn-PDI, (b, e, h) Zn-NDI, and (c, f, i) Zn-PMDI. The average film thickness is around 650 nm (inset in g–i) determined by the ImageJ program as shown in Figures S6, S8, and S10.

Zn-XDI MOFs were prepared from the three linkers as high-quality thin films on FTO, following a previously reported solvothermal protocol^30,35^ with minor adjustments (see General Methods and SI for details).^36^ The morphology of the surface-grown MOF thin films was examined by scanning electron microscopy (SEM), each displaying compact and homogeneous films (Figures 2g–i, S5, S7, and S9) with thicknesses ranging from a few hundred nanometers to one micrometer according to cross-section images. While the thicknesses can be tuned by precursor concentration and synthesis time, all experiments hereafter were performed on thin films of comparable thickness, around 650 nm (inset in Figure 2g–i; the film thickness was determined by the ImageJ program as shown in Figures S6, S8, and S10). To confirm the structural similarities of all three MOFs, thin film X-ray diffraction (XRD) was recorded (Figure 2d–f) and compared to the structural model published by Dincă and co-workers^35^ (the structural model for Zn-PDI and Zn-PMDI were built in analogy to that of Zn-NDI in Materials Studio software by performing geometry optimization using the Forcite tool). In line with the simulated structural models, two prominent diffraction peaks that correspond to (110) and (220) planes are shifted slightly to lower Bragg angles when going from Zn-PMDI to Zn-NDI but significantly for Zn-PDI (direct comparison of diffraction patterns is shown in Figure S11). This shift is consistent with the change in linker size for PMDI (14.2 Å) and NDI (14.8 Å) to 19.2 Å for the PDI linker (Figure 1).^37^ The consistency of each experimental diffraction pattern with the respective simulated structural model as well as the expected evolutionary diffraction trend in the isoreticular MOF series suggests that these Zn-XDI MOFs adopt a common structure type (with monoclinic C_2_ space group) where pyrazolate groups are bridged by an infinite chain of tetrahedral Zn^2+^ ions (Figure 2a–c). Moreover, XRD patterns of the thin films indicate a preferred orientation of the crystallites in the thin films, with the orthogonal channels being parallel to the conductive FTO surface.

### Electrochemistry of Isoreticular MOF Thin Films

**Figure 3:**
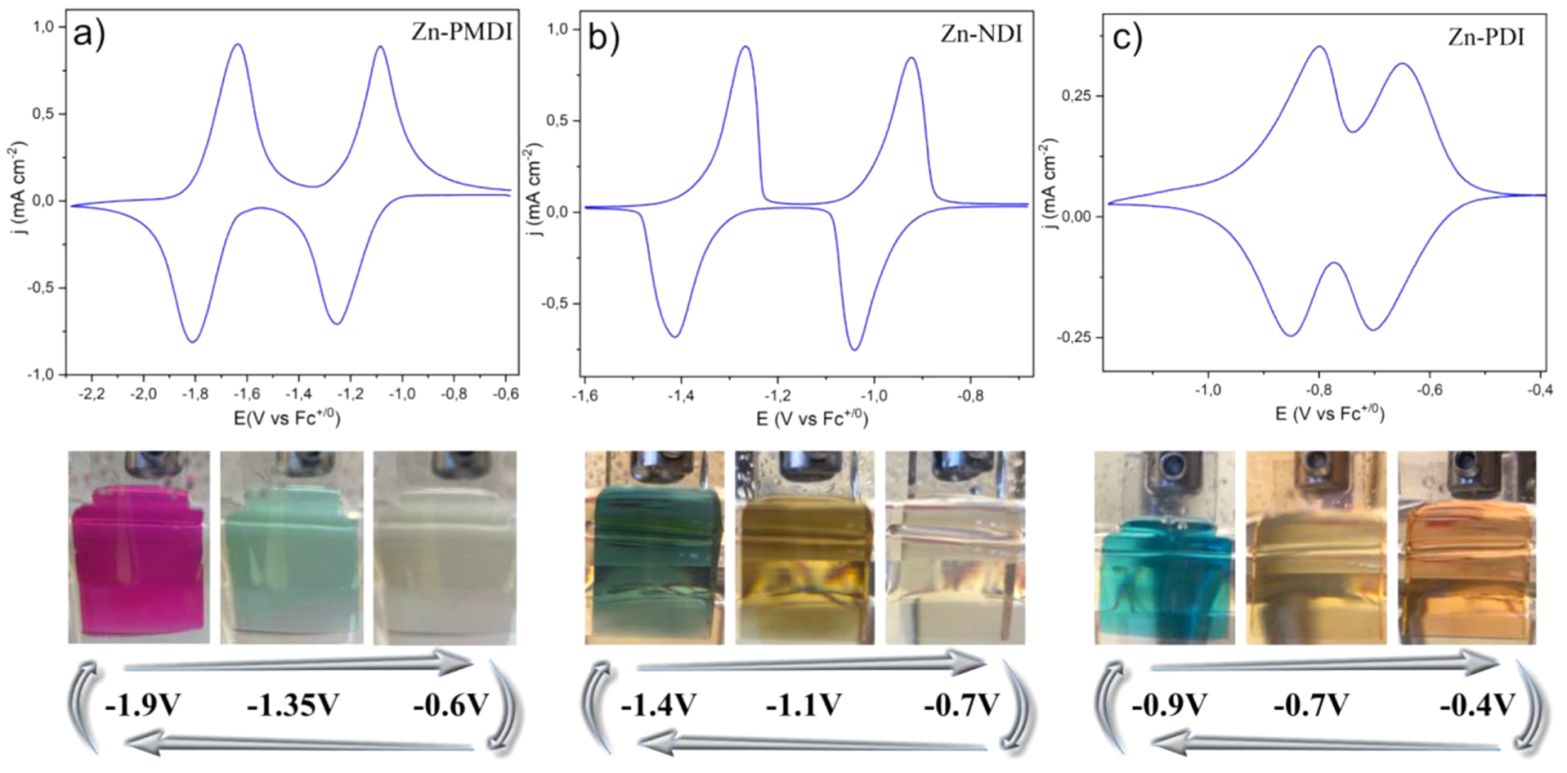
Representative cyclic voltammograms of Zn-XDI@FTO (X = PM, N, P) thin films, measured in DMF solution containing 0.5 M KPF_6_ at a scan rate ν = 25 mV s^–1^ (a) Zn-PMDI, (b) Zn-NDI, and (c) Zn-PDI. Bottom: photographs of the Zn-XDI@ FTO (X = PM, N, P) thin film electrodes displaying distinct colors in neutral, monoanionic, and dianionic states at indicated applied potentials.

The electrochemical properties of the Zn-XDI thin films on FTO were investigated by cyclic voltammetry (CV) in a three-electrode setup, using Zn-XDI@FTO as the working electrodes (see General Methods and SI for details). The CVs of all three Zn-XDI thin films show two reversible and normally ordered one-electron redox waves (Figure 3a–c). The observed formal redox potentials in the isoreticular MOF series correlate well with the corresponding values of the homogeneous linkers (Figures S18–S20 and summarized in Table S1) and are thus assigned to the linker-based [XDI]^0/•–^ and [XDI]^•–/2–^ redox couples. While the first reductions of the three Zn-XDI@FTO films are almost identical with their homogeneous counterparts within experimental error, the second reductions in the MOF films are consistently observed at somewhat milder potentials. This phenomenon has been observed previously and can be attributed to intermolecular interactions between the one-electron reduced linkers and the K^+^ counterion that facilitate the second reduction in the MOF.^38,39^ In other words, as compared to the homogeneous solution phase with separately solvated ions, ion pairing in the MOFs stabilize the electron accepting orbitals, thereby facilitating the subsequent reductions. The effect of the differing π-systems in the linkers of the isoreticular series manifests itself in the voltammetry response in two ways. First, the MOF with the largest π-system, i.e., Zn-PDI, exhibits the mildest potential for the first reduction at −0.67 V, followed by that of Zn-NDI and Zn-PMDI at −0.97 and −1.17 V, respectively. Furthermore, due to more extensive delocalization of the reduction in the largest π-systems, the [PDI]^•–/2–^ couple can be observed only 150 mV after the first reduction, a difference that increases during the series to 370 mV (difference between [NDI]^0/•–^ and [NDI]^•–/2–^) and 550 mV (difference between [PMDI]^0/•–^ and [PMDI]^•–/2–^). Consequently, the two redox waves in the CV of Zn-PDI@FTO are somewhat overlapping, while they are most separated in Zn-PMDI@FTO with the shortest linker (Figure 3a–c).

It is important to note that the individual XDI linkers in the Zn-XDI@FTO constructs are electronically isolated units. Therefore, electron transport through the materials proceeds by an electron hopping mechanism that is coupled to the translocation of charge-balancing counterions.^40−44^ Cation-coupled electron hopping transport is diffusional in nature and can be quantified by an apparent electron diffusion coefficient, *D*_e_^app^.^45−48^ An important difference to a homogeneous diffusion experiment is that electron diffusion by hopping from one immobilized redox-active site to the next has a physical boundary condition that is given by the finite thickness of the film (*d*_f_).^49^ As the time scale of a CV experiment can be altered by the scan rate (ν), two different diffusional regimes can be explored (Figures S21, S23, and S25). At fast scan rates, electron transport occurs in the semi-infinite regime, while slower scans experience the finite limit that is given by the film thickness. A dimensionless finite diffusion parameter (λ_e_) can be defined that correlates the film thickness to the thickness of the electron diffusional layer,^50,51^ according to eq 1
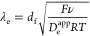
1with *R* and *T* being the universal gas constant and absolute temperature, respectively. Scan rate dependent CVs can be used to probe the boundary condition between the two limiting regimes, and a transition between the two behaviors will occur approximately when λ_e_ ≈ 1. As all Zn-XDI@FTO thin films have similar thicknesses, this transition scan rate will depend only on the *D*_e_^app^ values of the three materials. At slow scan rates, λ_e_ approaches zero, and the CV responses of all films are characteristic of symmetric “surface” waves (Figures S22a, S24a, and S26a) with peak currents showing a linear dependence on scan rate ν (Figures S22c, S24c, and S26c). On the contrary, when the scan rates are fast, λ_e_ approaches infinity, and the CV responses of the same MOF films exhibit classical diffusion waves (Figures S22b, S24b, and S26b). In this case, the peak current is proportional to the square root of ν (Figures S22d, S24d, and S26d). Rather unexpectedly, the scan rates at which the CVs transition from the finite to the semi-infinite regime are basically identical for all three MOF thin films at approximately 50 mV s^–1^. This is noteworthy, as *D*_e_^app^s are frequently shown to depend on the pore diameter of the material, with larger pores providing for more facile diffusion–migration of the accompanying counterions.^52^ With this notion in mind, the fastest *D*_e_^app^ would be expected for the MOF with the largest pore size of the series, i.e., Zn-PDI. As the thicknesses of the three films are very similar, identical transition scan rates require that the *D*_e_^app^ values of the three films are also very similar. To verify this prediction, the *D*_e_^app^s were independently determined by monitoring the absorbance change of XDI^•–^ (1st reduction of each Zn-XDI@FTO) in a potential-step chronoamperometry experiment (see the SI for details) by applying the modified Cottrell equation^53,54^ (eq 2)
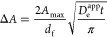
2where Δ*A* is the change in absorbance, *A*_max_ is the absorbance maximum, *t* is time in seconds, and *d* is the film thickness. The *D*_e_^app^’s of the three films were determined from the linear region of absorbance change versus square root of time and were found to be very similar in the range of 1 to 3 × 10^–10^ cm^2^ s^–1^ (Figure S28), consistent with the similar transition scan rates.

The reason for this unexpected finding may lie in the orientation of the crystallites in the thin film, with the 1-dimensional channels being parallel to the FTO surface. Charge propagation through the film is normal to the substrate surface, which implies that counterions have to diffuse orthogonal to the channel vector. We propose that this process is independent of the diameter of the channels and thus very similar for all three MOFs. The discussion is related to the finding that hopping charge transport in NU-1000 is anisotropic and orders of magnitude faster along the channels as compared to across the channels.^47^

### Spectroelectrochemistry of Isoreticular MOFs

**Figure 4:**
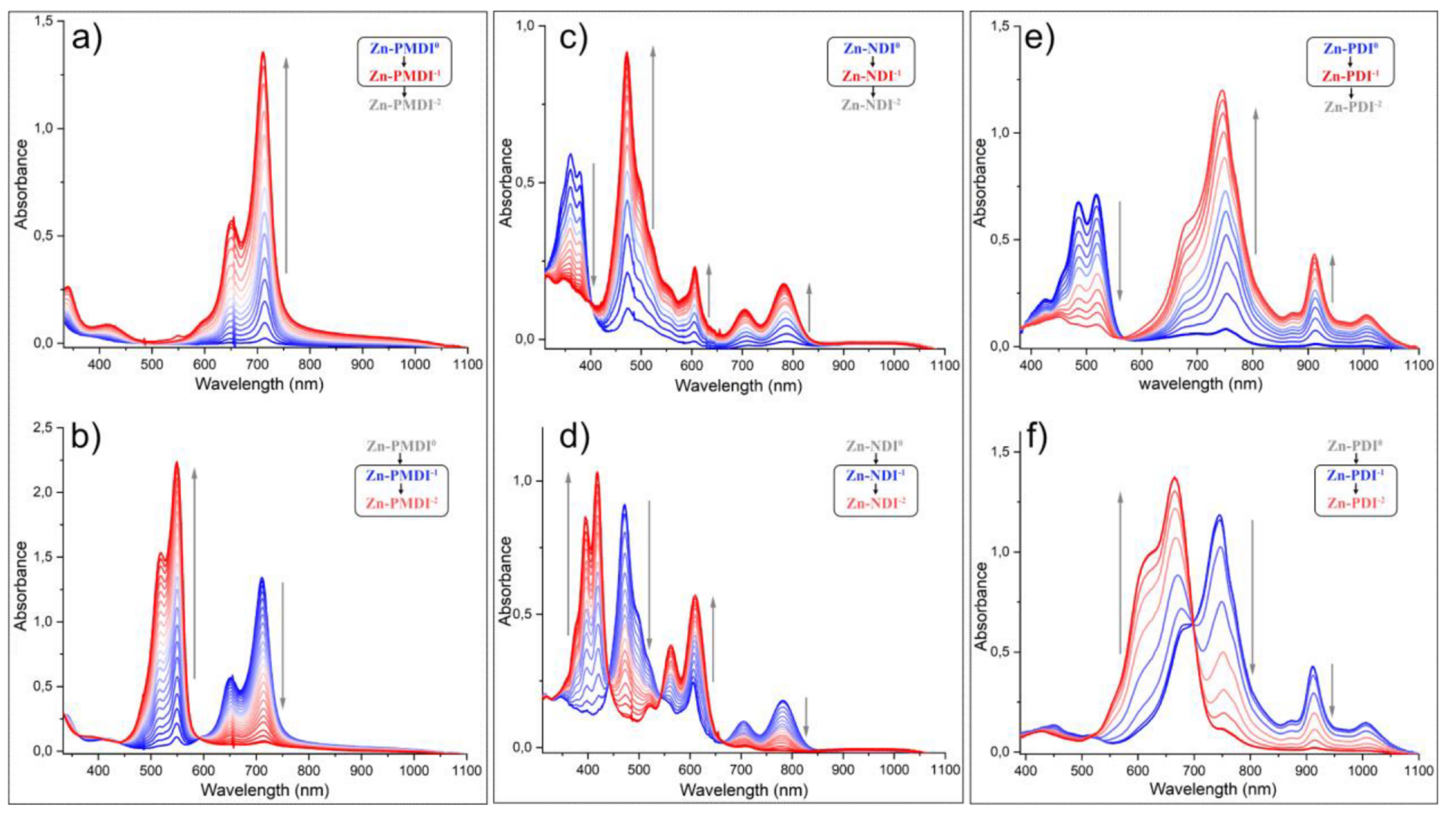
Changes in the electronic absorption spectra of Zn-XDI@FTO (X = PM, N, P) thin films as a function of applied potential. Spectroelectrochemical data of (a, b) Zn-PMDI, (c, d) Zn-NDI, and (e, f) Zn-PDI collected by transmission UV–vis spectroscopy show the formation of [PMDI]^•–^ and [PMDI]^2–^ when a double potential step chronoamperometry of (a) 0 → −1.35 V and (b) −1.35 → −1.9 V is applied and, similarly, formation of [NDI]^•–^ and [NDI]^2–^ at a double potential step of (c) 0 → −1.1 V and (d) −1.1 → −1.4 V and formation of [PDI]^•–^ and [PDI]^2–^ at double potential step of (e) 0 → −0.7 V and (f) −0.7 → −0.9 V (all potential vs Fc^+/0^).

The π-systems of varying sizes in the linkers of the isoreticular series give rise to different colors of the MOFs in the ground state. As expected, Zn-PDI with the largest π-linker has the lowest optical HOMO–LUMO gap and, thus, the most red-shifted absorption profile of the series, followed by that of the Zn-NDI and finally the Zn-PMDI, which is almost colorless (Figure S4). Each MOF exhibits distinct color changes upon cycling from neutral [XDI] to radical anion [XDI]^•–^ and dianion [XDI]^2–^ states. The changes in the oxidation state are induced by holding the applied potential at a value beyond the standard potential of the respective process, as specified at the bottom of Figure 3. Zn-PDI films switch from red to light green during the first reduction and further to blue after the second electron uptake. Zn-NDI switches from pale yellow to orange ([NDI]^•–^) and gray-blue ([NDI]^2–^), while Zn-PMDI films start almost colorless and switch to blue after the first reduction and pink at the [PMDI]^2–^ state (Figure 3, bottom panel). The electrochromism can be followed more quantitatively by UV–vis spectroelectrochemistry (see Figure S27, more details in the SI). As shown in Figure 4, all transformations are characterized by clearly visible isosbestic points, showing that the systems are well behaved and that no intermediate species accumulate on the time scale of the experiment. More specifically, ground state absorption maxima are observed at 485 and 519 nm for Zn-PDI, 359 and 379 nm for Zn-NDI, and 270 nm for Zn-PMDI. This significant blue shift of the electronic π–π* transitions in the neutral state correlates well with the apparent colors and again highlights the impact of the π–electron density in the diimide cores.^55^ The π–π* transitions of the MOFs are identical with those of the homogeneous linkers, as expected for MOFs with electronically isolated, redox-active linker units (Figures 4 and S4). This is a crucial feature in designing electrochromic materials with high coloration efficiency as it can maximize Faradaic transformations of the electrochromic units (see below for more details). For Zn-PDI thin films, upon the first electron reduction, the appearance of a main absorption peak at 746 nm can be assigned to the formation of [PDI]^•–^, which is concomitant with the disappearance of the π–π* band of neutral Zn-PDI (Figure 4e). When the applied potential is stepped beyond the second reduction, the absorption peak of [PDI]^•–^ at 746 nm decreases, and a set of new absorption peaks appear around 667 nm (Figure 4f), corresponding to the generation of [PDI]^2–^. Similarly, Zn-NDI and Zn-PMDI thin films undergo analogous absorption changes during the two one-electron-reduction processes (Figure 4a–d). In particular, [NDI]^•–^ and [NDI]^2–^ can be identified by their characteristic absorption bands at 472 and 418 nm^30,56^ and [PMDI]^•–^ and [PMDI]^2–^, at 714 and 549 nm, respectively.^57^ The distinct absorption features of these isoreticular MOF thin films are that they cover almost the whole range of the visible spectrum, which in turn promises multiple options for electrochromic displays. For simplicity and easy comparison, characteristic absorption bands of the isoreticular MOF series (free linkers as well) at different redox states are summarized in Table S2.

### Electrochromic Performances of Isoreticular MOFs

**Figure 5:**
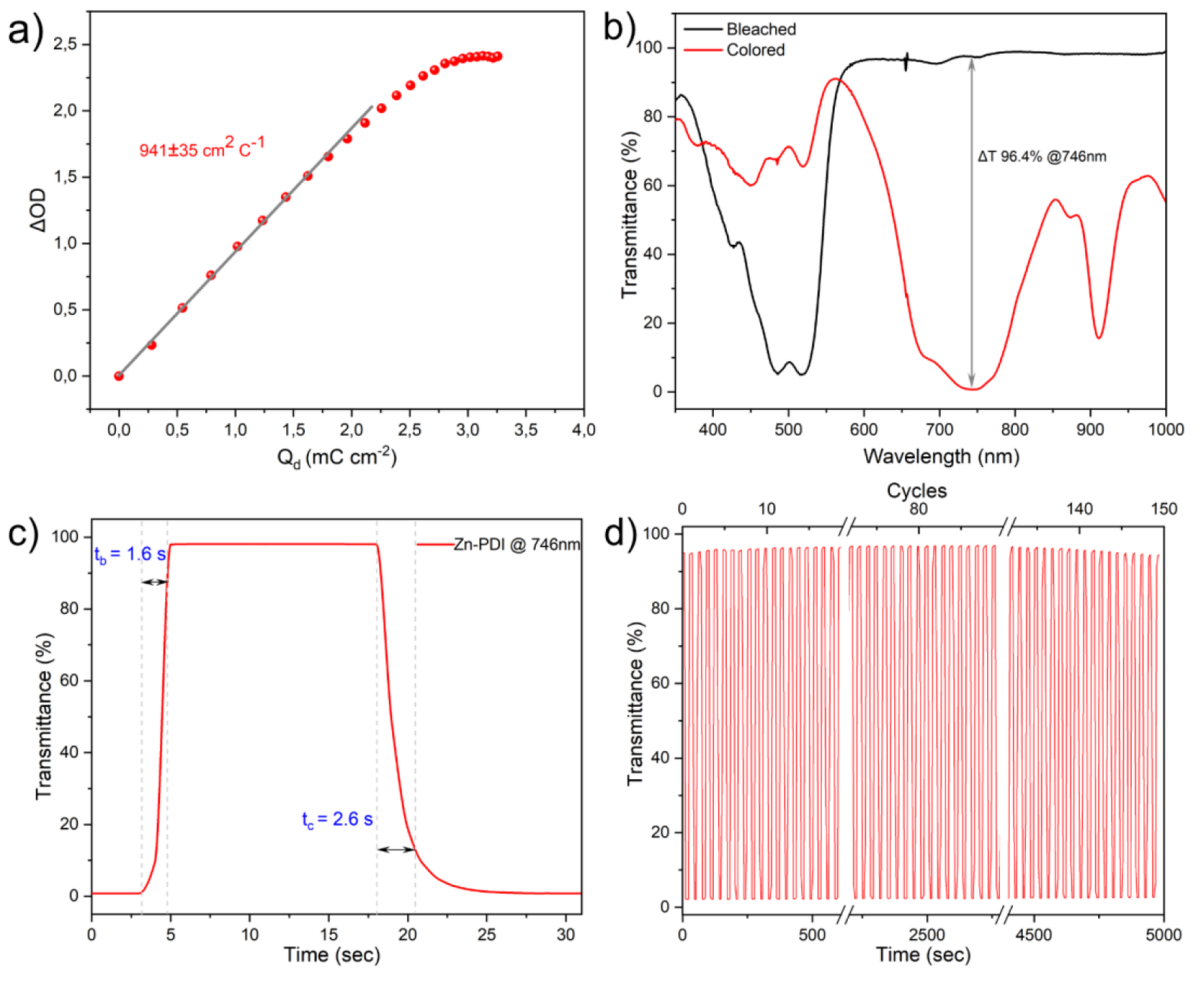
Electrochromic properties of Zn-PDI thin film monitored by spectroelectrochemistry. (a) The coloration efficiency of Zn-PDI at 746 nm, (b) comparison between the optical transmittance of the bleached and colored states, showing optical contrast Δ*T* > 96% at 746 nm, (c) fast switching time of 1.6 s for bleaching and 2.6 s for coloration (monitored at 746 nm), (d) stability of Zn-PDI@FTO thin film switching between 0 and −1.1 V (neutral and doubly reduced form) for 150 cycles. Optical transmittance of all MOF films was measured using a bare FTO plate in an electrolyte solution as the reference.

Encouraged by their easy preparation, uniform thin-film growth, and well-separated electrochemical and spectral features, the isoreticular MOFs were further evaluated for practical electrochromic applications. An important parameter in this context is the coloration efficiency (η, also known as electrochromic efficiency), which is a measure of the difference in absorptivity of two states, normalized to the charge that was required to induce the change.^58−62^ Coloration efficiencies for all processes in the isoreticular MOF series were determined using the following eq 3:
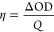
3where ΔOD is the change in optical density (between colored and bleached state) and *Q* is the corresponding charge density (C cm^–2^) used to induce the change in redox state (more details in the SI). Most notably, the η of the Zn-PDI film reaches 941 ± 35 cm^2^ C^–1^ at 746 nm (Figure 5a), which to the best of our knowledge, is a record-high value reported in MOFs (see Table S3).^28,63^ The other two thin films exhibit impressive coloration efficiency as well, with 610 ± 31 and 753 ± 46 cm^2^ C^–1^ for Zn-NDI (at 472 nm) and Zn-PMDI (at 714 nm), respectively (Figure S29). According to eq 3, the exceptionally high coloration efficiency is contributed by the high extinction coefficients of the organic diimide cores and a low charge density that is required to induce the color change. Compared to other XDI-based electrochromic materials, the latter acts as the primary factor for the record-high η as the redox-active XDI linkers are electronically isolated in the 3D porous MOF architecture. This characteristic feature maximizes Faradaic processes at each installed XDI unit and minimizes capacitive charge losses.

Second, the optical contrast (Δ*T*) which measures the degree of color change was quantified for all three thin films (Figures 5b, S30, and S31). Very high optical contrast (absorption change of up to 2.5 OD and Δ*T* up to 97%) is consistently obtained for all members of the isoreticular MOF series (measured at characteristic λ of [XDI]^0/•–^), which is among the highest in electrochromic materials.^59,64^ Meanwhile, the switching time between the bleached state and the colored state (90% of optical contrast) was determined for all thin films (Figures 5c and S32). Both coloration time and bleaching time are in the range of a few seconds (Figure 5c), demonstrating their practical feasibility.^65,66^ Lastly, the electrochromic stability of the isoreticular MOFs was investigated in accelerated reduction and oxidation operation cycles (details in the SI). The Zn-PDI MOF retains >99% of its initial electrochromic response over 100 oxidation/reduction cycles (Figure S33) and >98% over 150 cycles (Figure 5d). Zn-NDI films also show excellent electrochemical cyclability with <3% decrease in the peak absorbance over 50 cycles (Figure S34), while Zn-PMDI films are comparatively the least stable among the three, showing some degree of delamination upon excessive cycling. Furthermore, SEM and thin film XRD analyses were conducted to verify the morphological and structural integrity of MOF thin films after electrochemical operations (Figures S12–S17). Overall, the parameters of the isoreticular series are among the best in the field of electrochromic MOFs/COFs (Table S3) and are very competitive against conventional electrochromic materials.^67^ More importantly, our study highlights the fact that the modular property of MOFs brings numerous opportunities to design and tailor electrochromic materials upon demand, for example, by changing substituents at the diimide core, tuning the intermolecular interactions between neighboring linkers, and modulating the electronic coupling between the linker and metal nodes.

## Conclusions

In this study, we have designed and synthesized three dipyrazole-terminated XDI linkers that differ in their π-conjugated diimide cores and used them to construct a series of isoreticular Zn-XDI@FTO MOF thin films. The XDI linkers give rise to two reversible one-electron reductions in each of the MOFs that are anodically shifted as the conjugated π-systems in the XDI linkers are getting larger. Despite variations in pore apertures in the isoreticular MOFs, the scan rates at which the CVs of the three MOFs transition from finite to semi-infinite diffusion regimes are basically identical. As the Zn-XDI@FTO films are of similar thicknesses, this finding suggests that cation-coupled electron hopping transport is equally fast in all three materials. Supporting this notion, independently determined apparent electron diffusion coefficients, *D*_e_^app^, of all materials are indeed found to be very similar.

All MOF thin films exhibit distinct changes in their optical properties upon changing their redox state that can be monitored by operando UV–vis spectroscopy. The characteristic Faradaic electrochromism in the isoreticular series is attributed to the electronically isolated XDI linkers and differences in molecular orbital energy levels in their different oxidation states. Thin films of Zn-PDI display a high optical contrast of 96.4% at 746 nm and are stable over at least 150 oxidation/reduction cycles with <2% contrast attenuation. Furthermore, this material shows a rapid coloration time of 1.6 s and a bleaching time of 2.6 s and reaches a record electrochromic coloration efficiency of 941 C cm^–2^ at 746 nm. We believe that the presented study of an isoreticular MOF series with altering optical properties provides an appealing platform for further in-depth electrochemical analysis of redox-active MOFs, while also inspiring the design of electrochromic MOFs in the future.

## General Methods

### XDI (X = PM, N, P) Linkers and MOF Synthesis

XDIs (X = PM, N, P) were prepared by condensation of 2 equiv of 4-amino-3,5-dimethylpyrazole with the corresponding dianhydrides of XDI (X = PM, N, P); PMDI was refluxed in *N*,*N*-dimethylacetamide (DMA), NDI in *N*,*N*-dimethylformamide (DMF), and PDI in propionic acid following a modified procedure^35,68^ and characterized by NMR (see the Supporting Information for detailed synthetic procedures). The bulk MOFs were prepared solvothermally from a 1:1.1 molar mixture of XDI (X = PM, N, P) and Zn (NO_3_)_2_·6H_2_O in DMF at 130 °C for 4 h to afford the Zn-XDI MOFs as microcrystalline powders.

### Zn-(XDI)@FTO (X = PM, N, P) Thin Film Synthesis

FTO slides were cut into 2.5 × 1.1 cm^2^ pieces and cleaned by successive sonication in solutions of Alconox, ethanol, and acetone. A solution of Zn (NO_3_)_2_·6H_2_O (0.11 mmol) and XDI (0.10 mmol) in DMF was prepared in a 20 mL scintillation vial and sonicated for 10 min. After deaeration by bubbling with argon for 10 min, the precleaned FTO substrate was inserted into the reaction mixture. The vial was sealed and placed in a gravity convection oven for 4.5 h at 130 °C. The vials were allowed to cool to room temperature, and the films were washed with DMF and sonicated for 1 min to remove any loosely bound powder on the surface. The Zn-(XDI)@FTO slides were soaked in DMF for further use.

### Electrochemistry

Electrochemical analyses were performed in a standard three-electrode configuration connected to an Autolab PGSTAT204 potentiostat controlled with Nova 2.1.4 software. The electrode setup includes Zn-XDI@FTO (X = PM, N, P) thin films as the working electrode, glassy carbon as the counter electrode, and nonaqueous Ag/Ag^+^ as the reference electrode (10 mM AgPF_6_ in acetonitrile). Solutions of 0.5 M KPF_6_ in dry DMF were used as the supporting electrolyte. MOF-modified FTO was sonicated for 1 min to remove loosely bound partials prior to any experiments. The electrolytes were directly used as bought, and the solvent was taken from the solvent purification system (SPS) without any further purification. Before the experiment, argon was bubbled for 15 min in the electrolyte solution. The head space of the electrochemical cell was continuously purged with argon during the experiments. The applied potentials were calibrated against the ferrocene Fc^+/0^ redox couple.

### UV–vis Spectroelectrochemistry

UV–vis spectra of electrogenerated species were collected in situ using a diode array spectrophotometer (Agilent 8453) coupled to an Autolab PGSTAT100 potentiostat controlled with Nova 2.1.4 software. Redox processes were carried out in a homemade electrochemical cell, consisting of a quartz cuvette with 1 cm path length and a stopper designed to hold the three electrodes (Figure S27): Zn-XDI@FTO thin film as the working electrode, a Pt rod as the counter electrode, and nonaqueous Ag/Ag^+^ as the reference electrode (10 mM AgPF_6_ in acetonitrile), with only the working electrode in the optical path.

## Supporting Information Available

Synthesis procedures and NMR spectroscopic characterizations of the linkers, comparison of UV–vis absorption spectra and CVs of linkers and Zn-XDI (X = PM, N, P) MOF thin films on FTO, electrochemical (EC) cycling stability, comparison of crystallinity through XRD and SEM of MOF thin films before and after EC cycling, scan rate dependent CVs of MOFs and their transition from finite to semi-infinite diffusion regime, schematic illustration of SEC setup, *D*_e_^app^ data, and electrochromic performances: CE, optical contrast, switching time and stability of Zn-XDI (X = PM, N, P) MOF thin films, and comparisons with previously reported MOF-based electrochromic materials (PDF)
Video showing coloration and bleaching processes of Zn-PDI@FTO upon electrochemical cycling (0.5 M KPF_6_ electrolyte solution) (MP4)
