# Supplementary material for: Electrochromism in Isoreticular Metal–Organic Framework Thin Films with Record High Coloration Efficiency
Source: ACS Nano. 2023 Oct 18;17(21):21595–603. doi: 10.1021/acsnano.3c06621 (PMC10655172; doi:10.1021/acsnano.3c06621)
Supplement: Supplementary file 1 — nn3c06621_si_001.pdf [file nn3c06621_si_001.pdf]

Supporting information

for

Electrochromism in Isoreticular Metal-Organic Framework Thin Films with Record High Coloration Efficiency

Amol Kumar,[a] *Jingguo Li,*[a],* *A. Ken Inge,*[b] *Sascha Ott*[a],*

[a] Department of Chemistry - Ångström Laboratory, Uppsala University, Box 523, 75120 Uppsala, Sweden.

[b] Department of Materials and Environmental Chemistry, Stockholm University, 106 91 Stockholm, Sweden.

E-mail: sascha.ott@kemi.uu.se; jingguo.li@kemi.uu.se

Contents

General information: Materials and Instrumentation 3
Synthesis and characterization of linkers 4
  Thin film formation 9
Thin-film characterization 9
  Scanning electron microscopy 9
  Thin film X-ray diffraction 11
  Stability of Zn-XDI MOF thin-films after EC cycles 12
Electrochemistry 15
  Cyclic voltammetry of linkers 15
  Cyclic Voltammetry of MOF Thin Film 15
  CVs of MOF thin films; transition from finite to semi-infinite diffusion regime 17
Spectroelectrochemical analysis (SEC) 20
  Apparent diffusion coefficients 22
Electrochromic performances 23
  Coloration efficiency and extracted charge 23
  Optical contrast 24
  Switching time 25
  Spectroelectrochemistry stability test 26
References 28

General information: Materials and Instrumentation

Materials

All solvents and commercially supplied chemicals were reagent grade and used as received without further purification unless stated otherwise. Zn $(NO_3)_2 \cdot 6H_2O$ (Sigma-Aldrich), benzene-1,2,4,5-tetracarboxylic dianhydride (≥99.0%) from Sigma-Aldrich, naphthalene-1,4,5,8-tetracarboxylic dianhydride (≥97.0%) from TCI, 1,6,7,12-tetrachloroperylene-3,4,9,10-tetracarboxylic dianhydride (≥95.0%) and 4-amino-3,5-dimethylpyrazole (≥95.0%) were purchased from Fluorochem. Potassium hexafluorophosphate (KPF_6, for electrochemical analysis, ≥99.0%) and fluorine-doped tin oxide (FTO) substrates (7 Ω/sq) were purchased from Sigma-Aldrich. N, N dimethylacetamide (DMA) (99.9%) and N, N-dimethylformamide (DMF) (99.9%) were purchased from VWR.

Nuclear Magnetic Resonance (NMR): ^{1}H NMR spectra were measured using a JEOL 400 MHz spectrometer at 293 K. Proton chemical shifts are expressed in parts per million (δ scale) and are calibrated using residual non-deuterated solvent peaks as an internal reference (^{1}H NMR: $CDCl_3$: 7.26, DMSO-d^6: 2.50). Data for ^{1}H NMR spectra are reported in the following way: chemical shift (δ ppm) (multiplicity, integration). Multiplicities are reported as follows: s = singlet, d = doublet, t = triplet, q = quartet, m = multiplet.

Thin film X-Ray Diffraction (PXRD): Thin film XRD patterns were collected using a Siemens D5000 Kristalloflex equipped with a Gobel mirror (Bruker) and a parallel-plate collimator (0.40°) using a monochromatic Cu Kα radiation (λ = 1.5406 Å) source operating at 45 kV and 40 mA. Thin film XRD data was collected in theta to theta mode between 3 and 26° (2θ) range at a step size of 0.02°.

Scanning Electron Microscopy (SEM): Scanning electron microscopy (SEM) images were obtained using a Zeiss 1550 Schottky field emission scanning electron microscope equipped with an in-lens detector operated at 1 – 30 kV acceleration voltage. MOF thin films were anchored to conductive carbon tape on a sample holder disk. The surface area and film thickness of each individual film were determined using ImageJ[1].

Synthesis and characterization of linkers

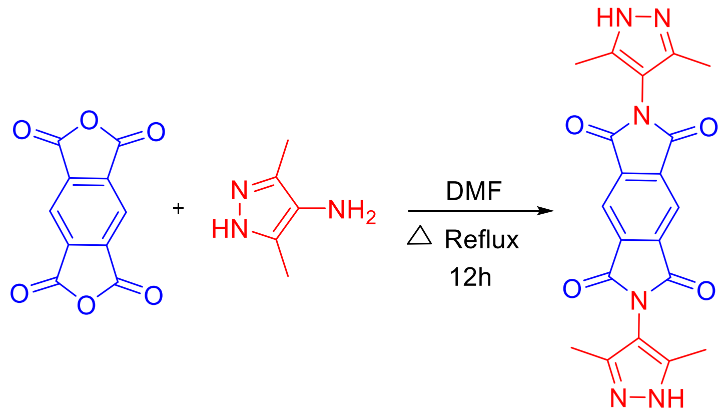

A dry 50 mL Schlenk round bottom flask was charged with pyromellitic dianhydride (0.5 g, 2.29 mmol) and 4-amino-3,5-dimethylpyrazole (1.1 g, 5 mmol) in anhydrous N, N dimethylacetamide (25 ml) under argon atmosphere. The resulting dark reaction mixture was refluxed at 130 °C with rapid stirring for 8 hrs. After cooling to room temperature, the orange solution was filtered and washed with successive aliquots of dichloromethane and dried under a vacuum. The resulting lemon-yellow solid was then recrystallized from DCM to give 0.78g (84%) bright yellow crystalline powder. ^{1}H NMR (400 MHz, DMSO-d_6) δ ppm: 12.61 (s, 2H), 8.37 (s, 4H), 2.06 (s, 12H).

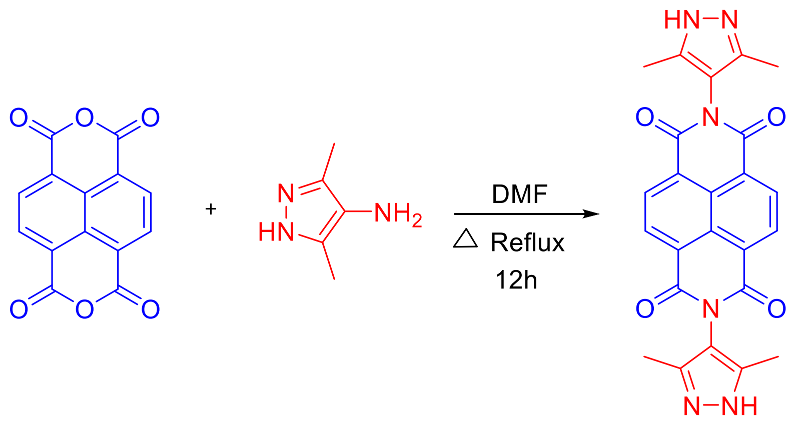

A dry 100 mL Schlenk round bottom was charged with 1,4,5,8 naphthalene tetracarboxylic dianhydride (0.86 g, 3.2 mmol), 4-amino-3,5-dimethylpyrazole (0.75 g, 6.8 mmol), and anhydrous DMF (50 mL) under Argon atmosphere. The reaction mixture was refluxed at 140 °C with rapid stirring for 12 hrs. The flask was cooled to room temperature and the dark brown DMF solution was poured into diethyl ether (150 mL) under stirring. The yellow precipitate was collected by filtration and recrystallized from DMF/diethyl ether (10 mL: 20 mL). The product was filtered and dried in vacuo at 70 °C to afford 1.05g (72%) yellow crystalline powder.[2] ^{1}H NMR (400 MHz, DMSO-d_6) δ ppm: 12.50 (s, 2H), 8.74 (s, 4H), 2.02 (s, 12H).

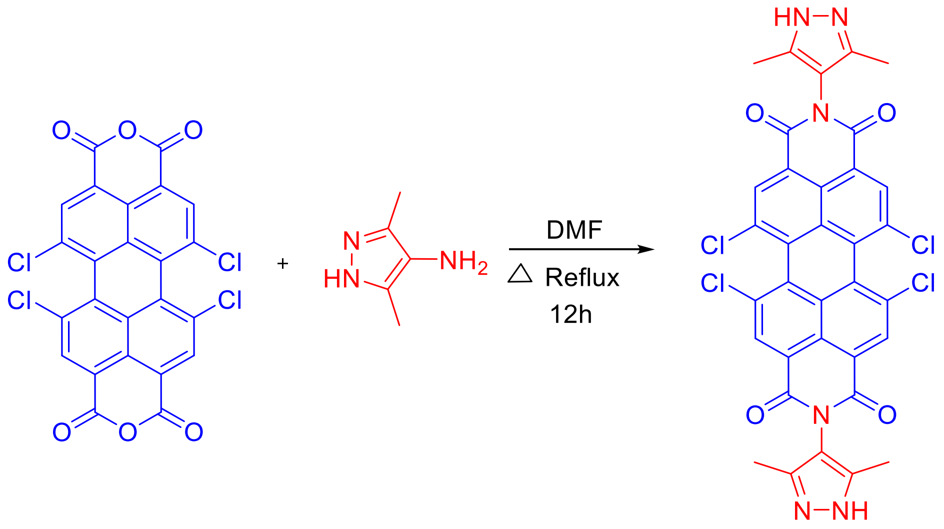

4-Amino-3,5-dimethylpyrazole (1.048 g, 9.44 mmol) was added to a mixture of 1,6,7,12-tetrachloroperylene-3,4,9,10-tetracarboxylic dianhydride (0.996 g, 1.88 mmol) and propionic acid (20 mL) under an argon atmosphere. The reaction mixture was stirred and heated at 140 °C for 24 h. After cooling to room temperature, the resulting precipitate was filtered and washed with deionized water until the filtrate became neutral. The crude product was then extracted with dichloromethane (DCM) and dried in vacuo to give 1.05g (78%) product as a red solid. ^{1}H NMR (400 MHz, DMSO-d_6) δ ppm: 12.39 (s, 2H), 8.63 (s, 4H), 2.04 (s, 12H).

^{1}H NMR spectra of XDI: Non-deuterated solvent peaks and H_2O/H_DO are marked with asterisks.

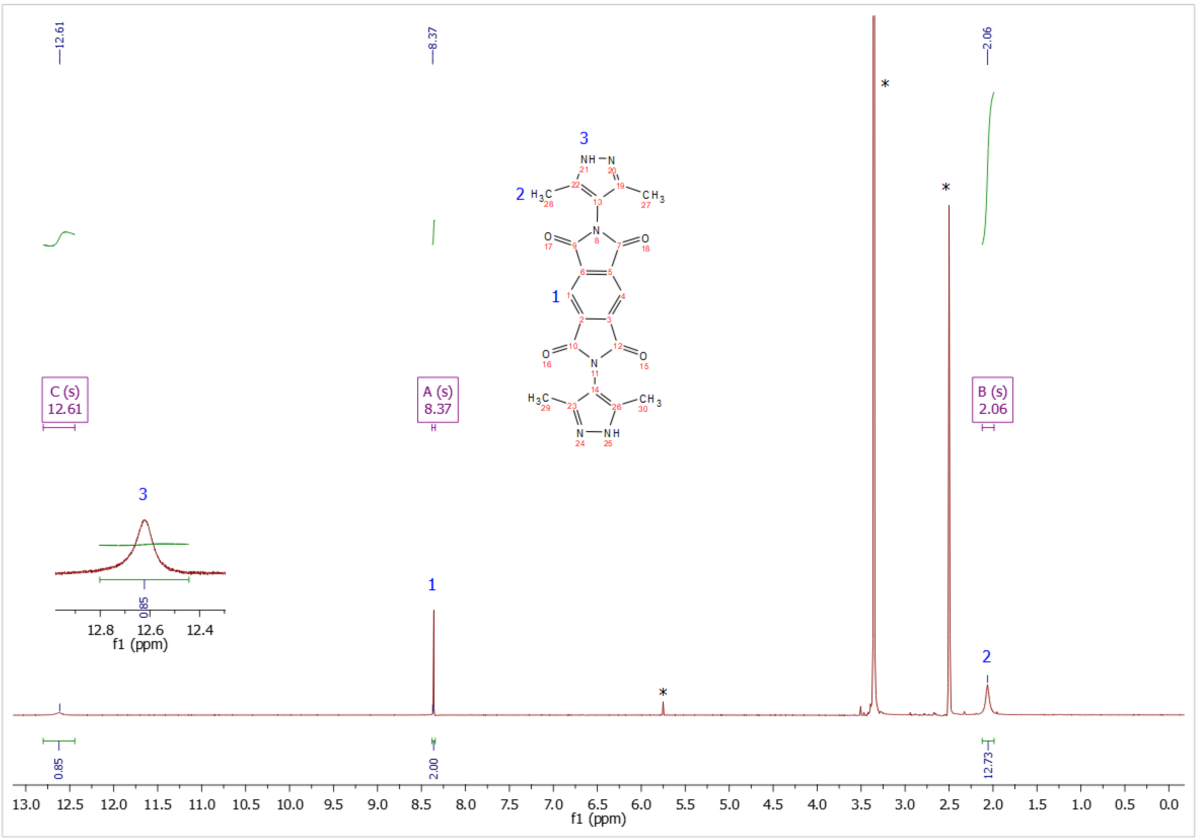

Figure S1: ^{1}H NMR spectrum of **PMDI** in DMSO-d6 at 293 K. Peak at 3.33 ppm corresponds to water, 5.63 ppm corresponds to DCM.

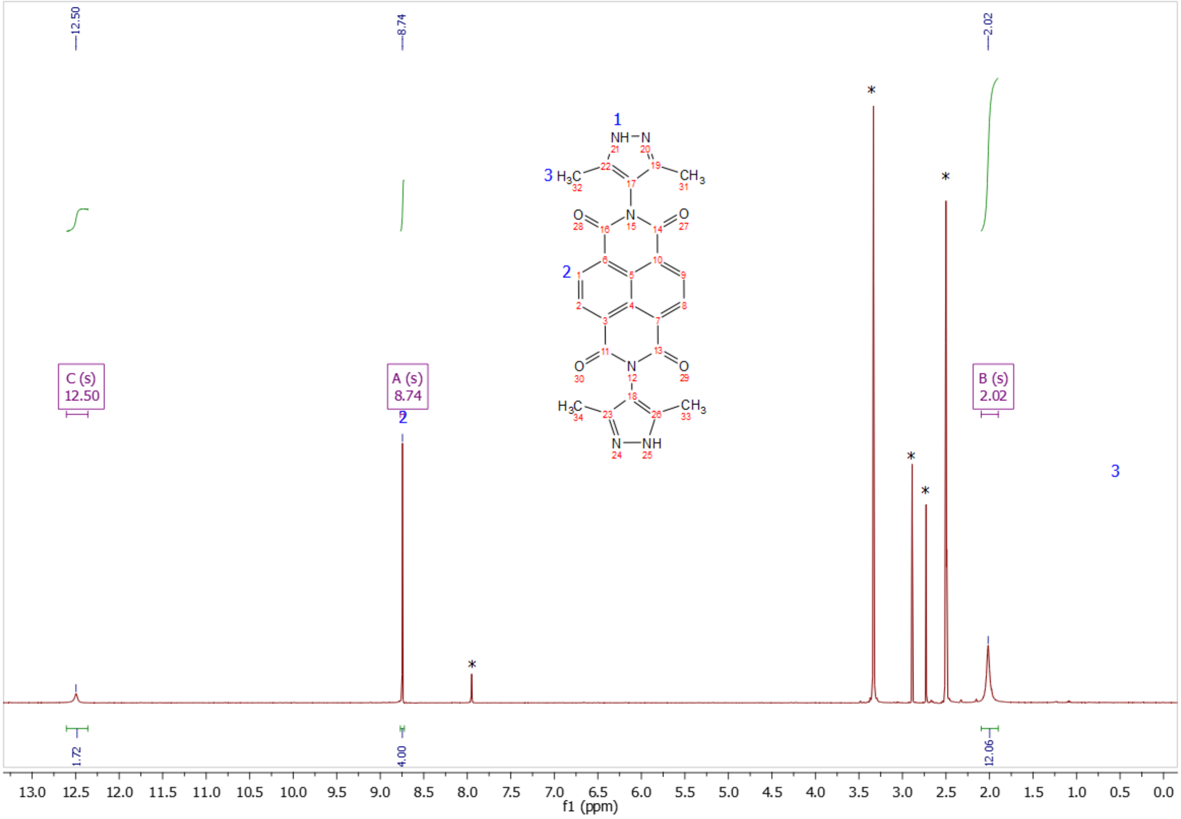

Figure S2: ^{1}H NMR spectrum of **NDI** in DMSO-d6 at 293 K. Peak at 3.33 ppm corresponds to water, and 2.7, 2.8, and 7.9 ppm corresponds to DMF.

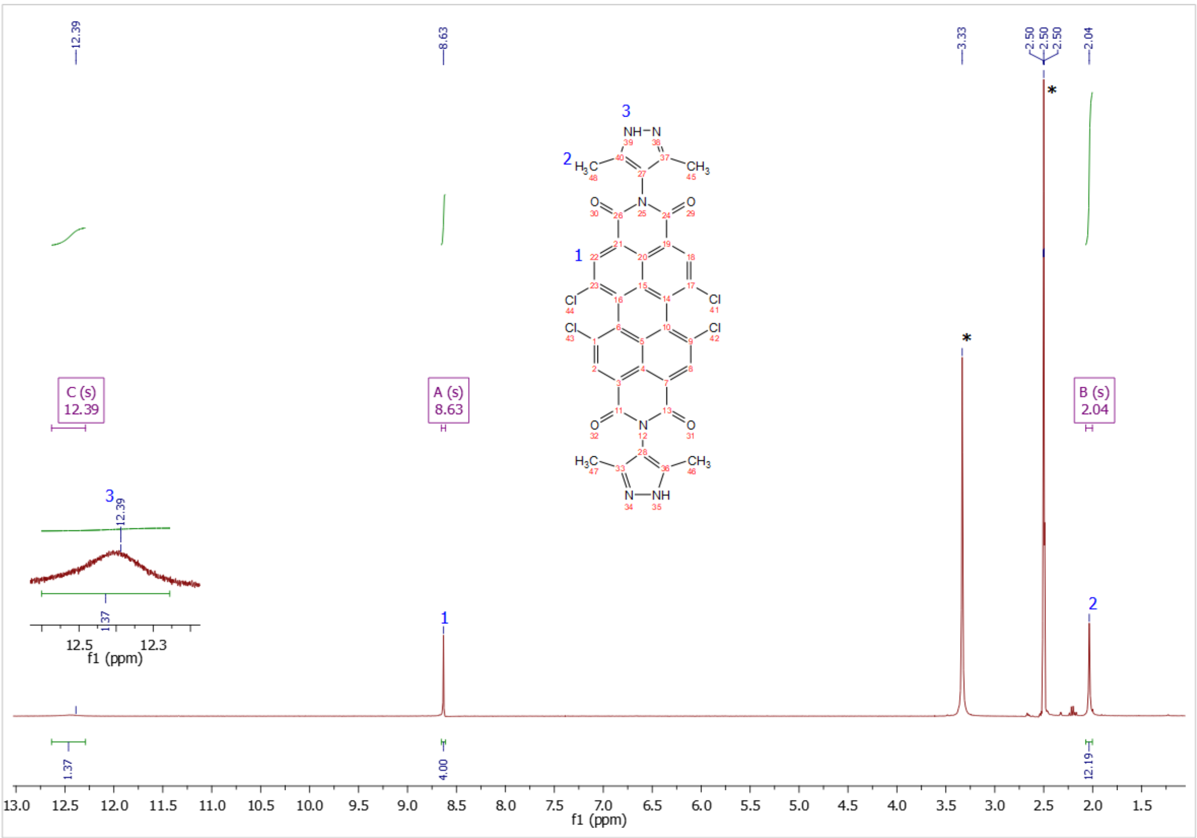

Figure S3: ^{1}H NMR spectrum of **PDI** in DMSO-d6 at 293 K. Peak at 3.33 ppm corresponds to water.

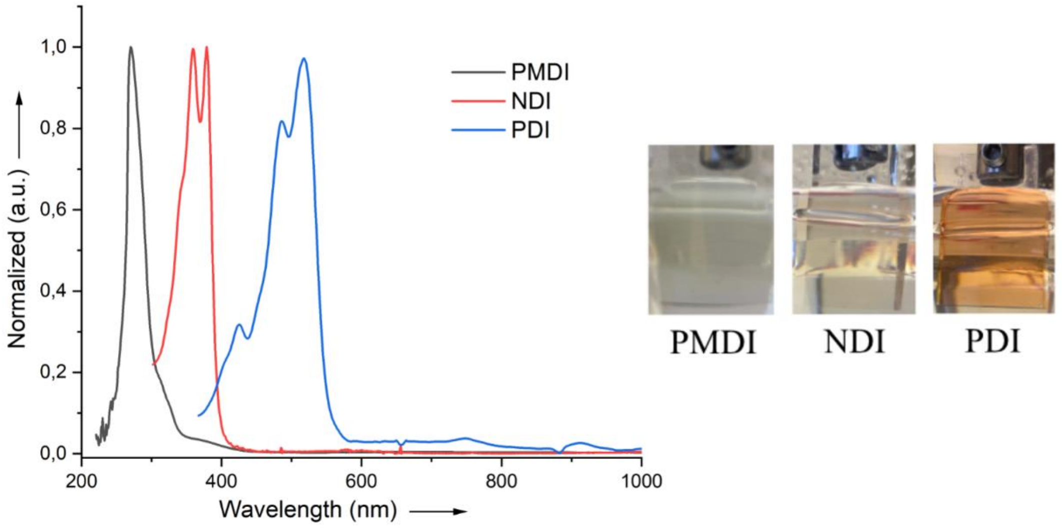

Figure S4: UV/Vis absorption spectra of solutions of XDI (X = PM, N, P) linkers in DMF, showing that the electronic π−π* transitions in the neutral states correlate well with the apparent colors of the corresponding as synthesized Zn-XDI@FTO MOF thin films. UV/Vis absorption of XDI in solution is measured with DMF as a reference to avoid any possible contribution from DMF.

Thin film formation

FTO slides were cut into 2.5 × 1.1 cm^2 pieces and cleaned by successive sonication in solutions of Alconox (1%), ethanol, and acetone. Simultaneously, a solution of Zn $(NO_3)_2{\cdot}6H_2O$ (0.11 mmol) and XDI (0.10 mmol) in DMF was prepared in a 20 mL scintillation vial and sonicated for 10 min. After deaeration with argon for 10 min, the precleaned FTO slides were inserted into the reaction mixture with the FTO side facing down. The vial was sealed and placed in a gravity convection oven for 4.5 hours at 130°C. The vials were allowed to cool to room temperature, and the films were washed with DMF and sonicated for 1 min to remove any loosely bound powder on the surface. The Zn-XDI@FTO slides were soaked in DMF until further use.

Thin-film characterization

Scanning electron microscopy

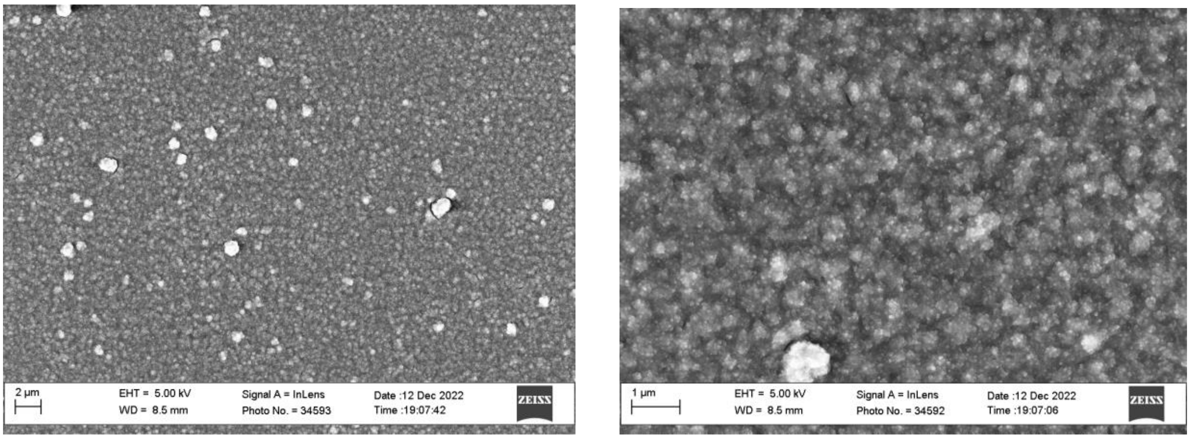

Figure S5: SEM image of Zn-PMDI@FTO thin films: low magnification (left), high magnification (right).

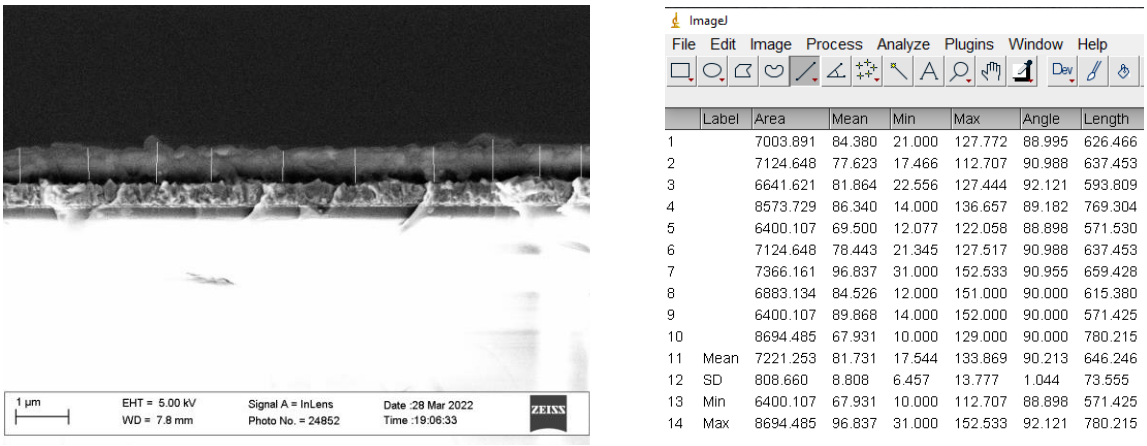

	Label	Area	Mean	Min	Max	Angle	Length
1		7003.891	84.380	21.000	127.772	88.995	626.466
2		7124.648	77.623	17.466	112.707	90.988	637.453
3		6641.621	81.864	22.556	127.444	92.121	593.809
4		8573.729	86.340	14.000	136.657	89.182	769.304
5		6400.107	69.500	12.077	122.058	88.898	571.530
6		7124.648	78.443	21.345	127.517	90.988	637.453
7		7366.161	96.837	31.000	152.533	90.955	659.428
8		6883.134	84.526	12.000	151.000	90.000	615.380
9		6400.107	89.868	14.000	152.000	90.000	571.425
10		8694.485	67.931	10.000	129.000	90.000	780.215
11	Mean	7221.253	81.731	17.544	133.869	90.213	646.246
12	SD	808.660	8.808	6.457	13.777	1.044	73.555
13	Min	6400.107	67.931	10.000	112.707	88.898	571.425
14	Max	8694.485	96.837	31.000	152.533	92.121	780.215

Figure S6: SEM cross-section image of Zn-PMDI@FTO thin film (left). ImageJ program was used to calculate the average thickness of the film (right).

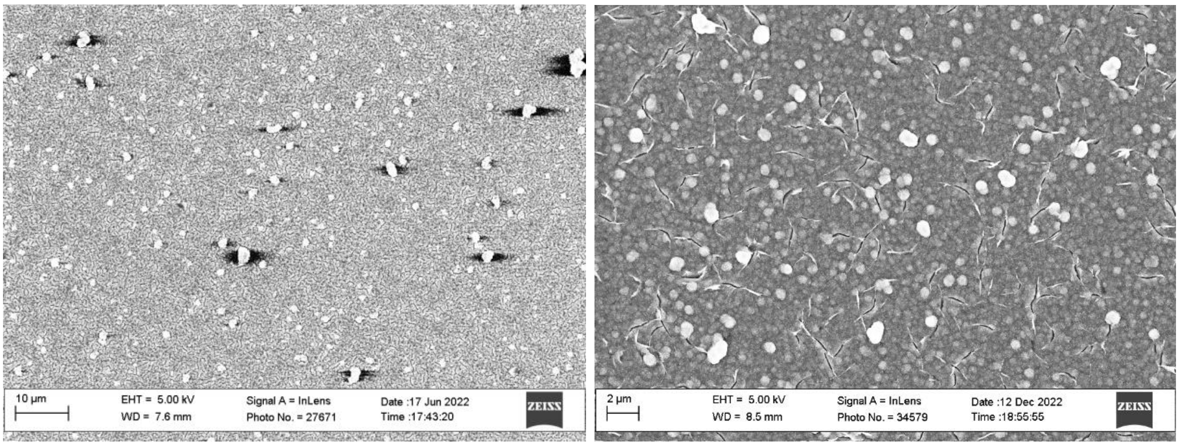

Figure S7: SEM image of Zn-PDI@FTO thin film. low magnification (left), High magnification (right).

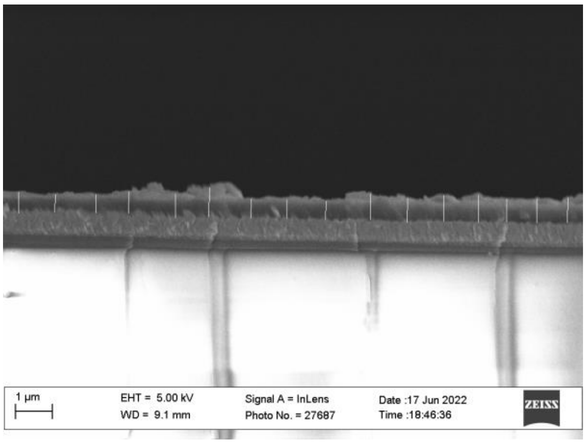

ImageJ

File Edit Image Process Analyze Plugins Window Help

	Label	Area	Mean	Min	Max	Angle	Length
1		10872.775	215.953	173.864	255.000	88.698	684.114
2		10389.541	95.977	60.000	134.000	90.000	652.850
3		9906.306	93.537	68.000	128.000	90.000	621.762
4		11597.627	97.062	66.000	132.000	90.000	730.570
5		11114.392	93.391	65.000	122.000	90.000	699.482
6		10389.541	94.049	70.190	132.333	88.636	653.035
7		11114.392	89.022	64.000	124.000	90.000	699.482
8		7973.368	79.723	61.750	109.781	88.210	497.652
9		8456.603	83.429	67.000	113.000	90.000	528.497
10		8456.603	85.573	69.529	112.000	88.315	528.726
11		12564.096	94.235	67.059	122.882	88.877	792.898
12		10389.541	84.093	63.000	128.000	90.000	652.850
13		9423.072	80.564	60.711	113.211	88.493	590.878
14		7973.368	82.606	59.000	113.000	90.000	497.409
15		9181.455	83.000	60.000	112.000	90.000	575.130
16		7731.751	79.839	59.839	114.968	88.152	482.116
17	Mean	9845.902	95.753	70.934	129.136	89.336	617.966
18	SD	1451.045	32.638	27.689	34.583	0.796	93.319
19	Min	7731.751	79.723	59.000	109.781	88.152	482.116
20	Max	12564.096	215.953	173.864	255.000	90.000	792.898

Figure S8: SEM cross-section image of Zn-PDI@FTO thin film (left). ImageJ program was used to calculate the average thickness of the film (right).

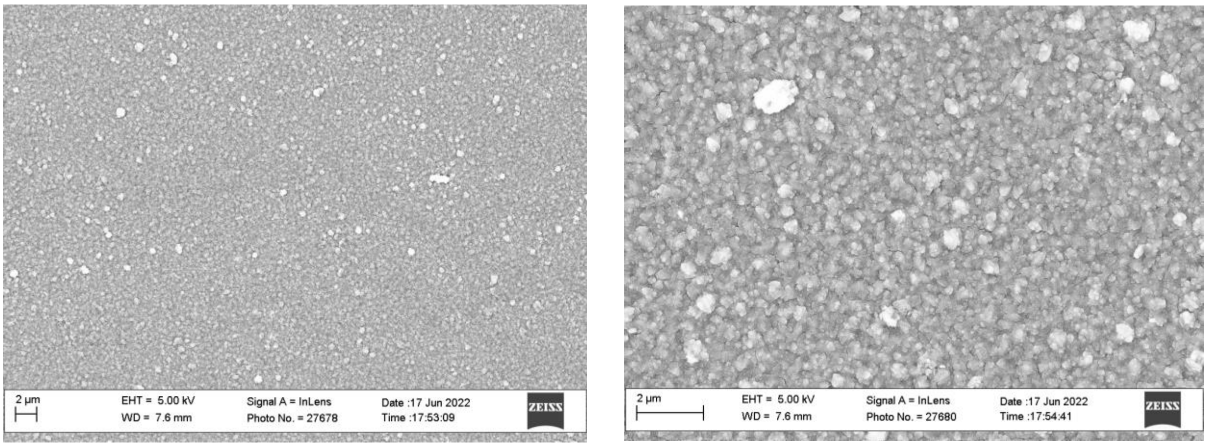

Figure S9: SEM image of Zn-NDI@FTO thin film: low magnification (left), High magnification (right).

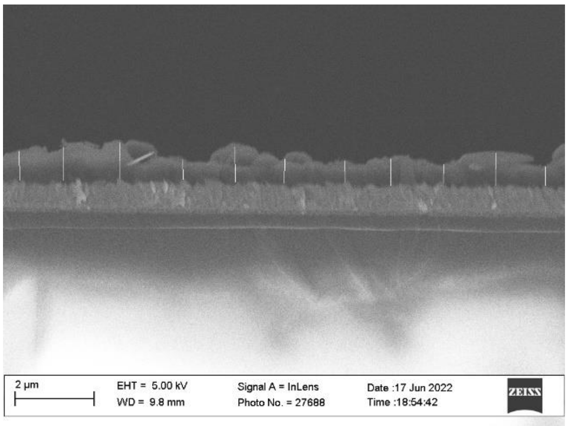

File Edit Font

	Label	Area	Mean	Min	Max	Angle	Length
1		10465.046	218.855	180.434	255.000	91.081	737.950
2		6782.900	99.343	85.000	122.000	90.000	473.318
3		11627.829	102.099	82.034	122.136	89.029	821.463
4		6782.900	100.384	86.176	121.676	88.315	473.522
5		9496.060	98.898	83.000	121.000	90.000	668.213
6		7558.089	105.933	90.316	124.632	91.507	529.185
7		8720.872	104.846	81.114	129.886	88.698	612.687
8		12596.814	104.189	83.719	126.828	90.895	891.059
9		7170.494	218.170	179.917	255.000	91.591	501.353
10		12015.423	102.968	84.000	128.000	90.000	849.187
11		12984.409	108.260	84.379	129.667	90.868	918.898
12	Mean	9654.621	123.995	101.826	148.711	90.180	679.712
13	SD	2404.675	46.815	38.811	52.648	1.125	172.711
14	Min	6782.900	98.898	81.114	121.000	88.315	473.318
15	Max	12984.409	218.855	180.434	255.000	91.591	918.898

Figure S10: SEM cross-section image of Zn-NDI@FTO thin film (left). ImageJ program was used to calculate the average thickness of the film (right).

Thin film X-ray diffraction

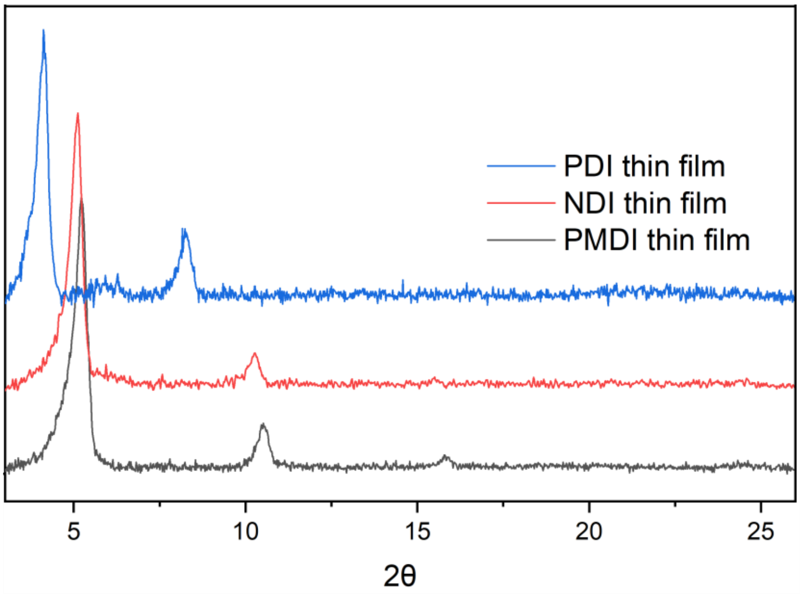

Figure S11: Thin film X-ray diffraction patterns (parallel beam theta to theta measurements) for Zn-XDI@FTO (X=PM, N, P), showing peak shifts to lower Bragg angles when going from Zn-PMDI to Zn-PDI.

Stability of Zn-XDI MOF thin-films after EC cycles

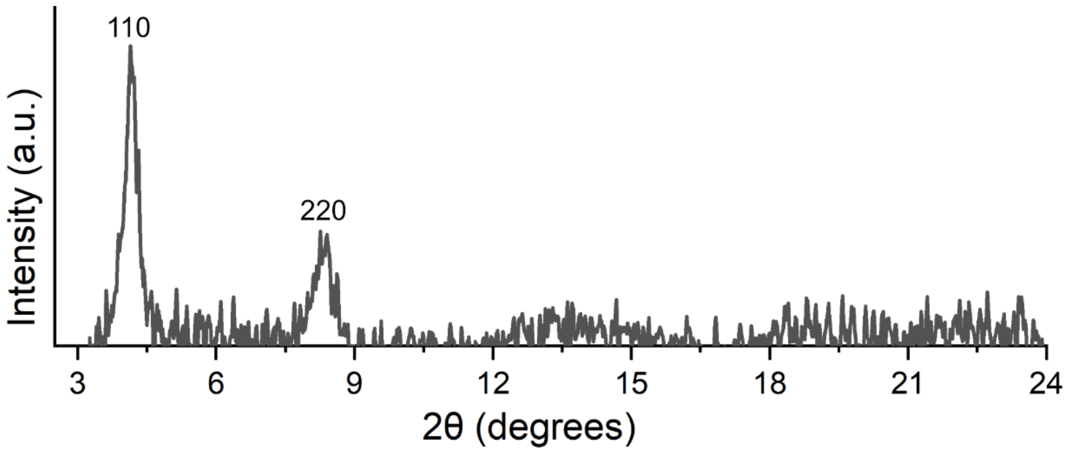

Figure S12: Thin-film XRD of Zn-PDI on FTO surface after 100 CV cycles stability test in DMF with 0.5 M KPF_6 as the supporting electrolyte.

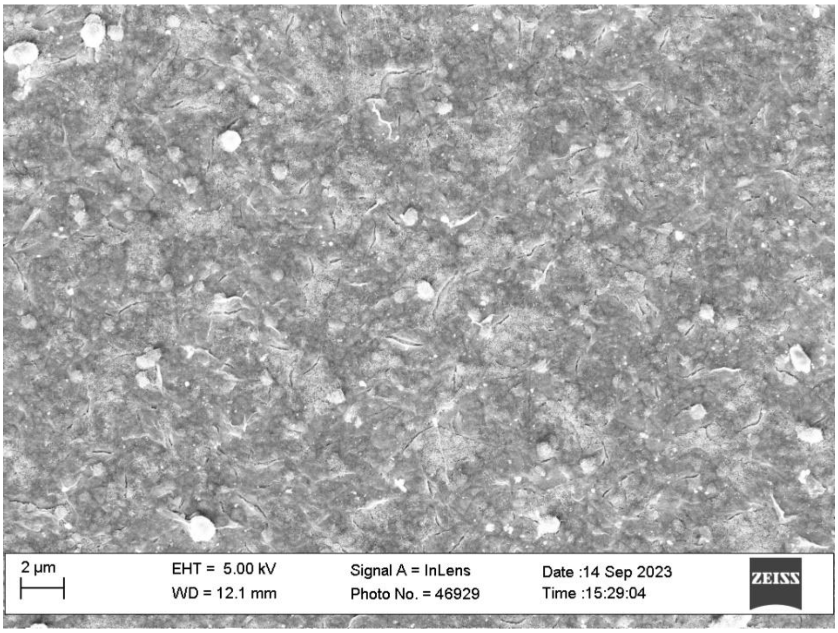

Figure S13: SEM top surface image of Zn-PDI on FTO surface after 100 CV cycles stability test in DMF with 0.5 M KPF_6 as the supporting electrolyte.

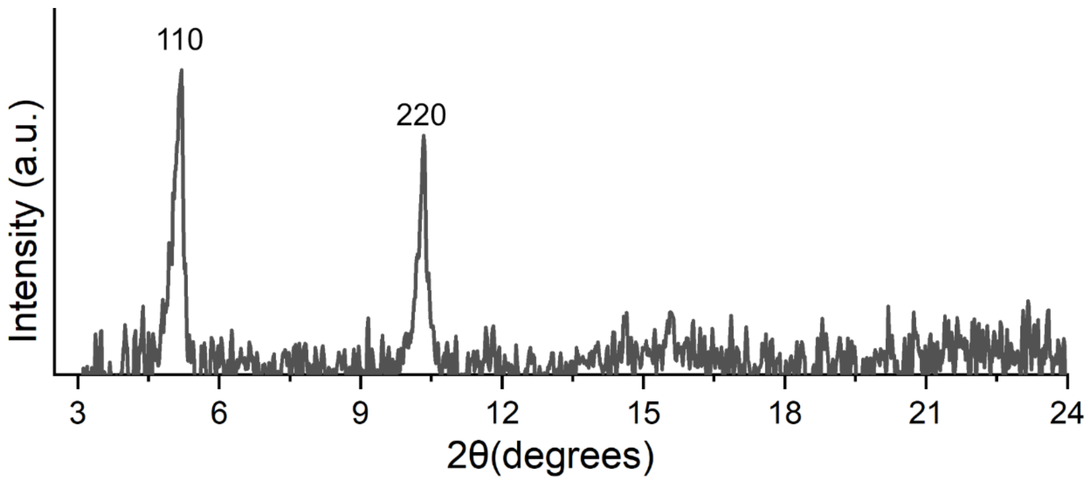

Figure S14: Thin-film XRD of Zn-NDI on FTO surface after 100CV cycles stability test in DMF with 0.5 M KPF_6 as the supporting electrolyte.

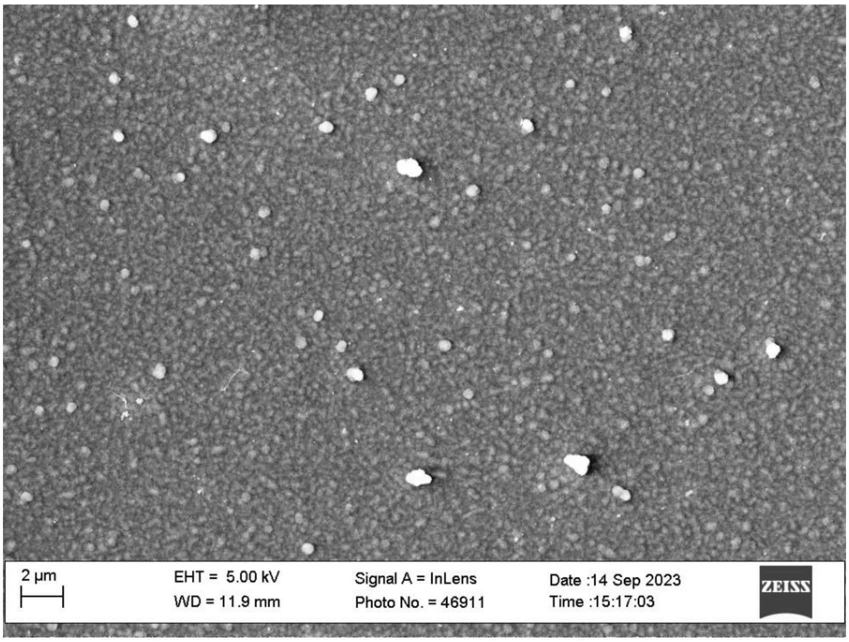

Figure S15: SEM top surface image of Zn-NDI on FTO surface after 100 CV cycles stability test in DMF with 0.5 M KPF_6 as the supporting electrolyte.

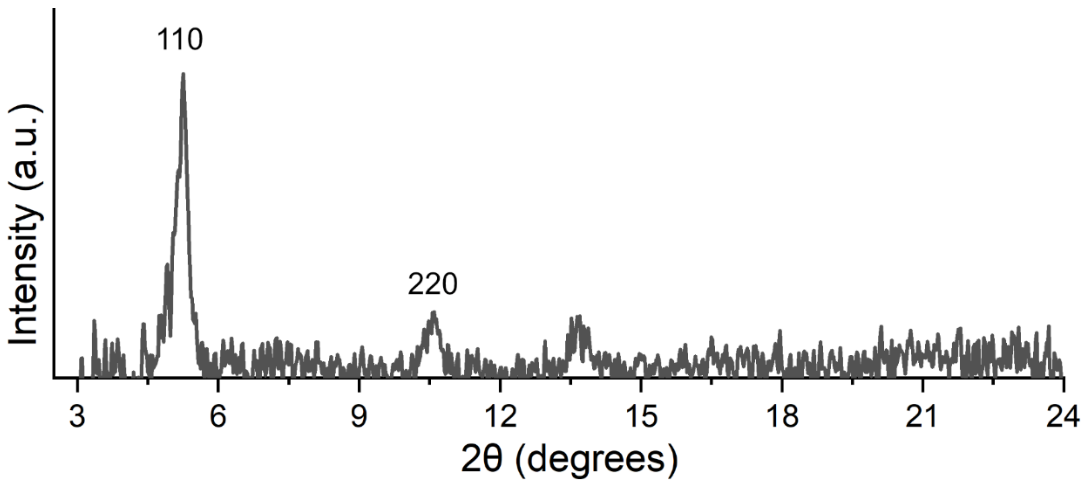

Figure S16: Thin-film XRD of Zn-PMDI on FTO surface after 25 CV cycles stability test in DMF with 0.5 M KPF_6 as the supporting electrolyte.

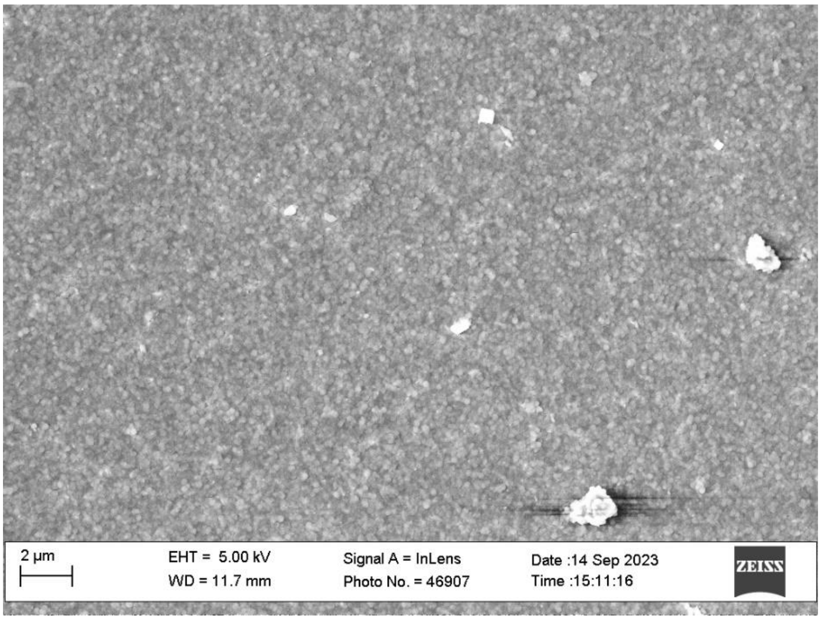

Figure S17: SEM top surface image of Zn-PMDI on FTO surface after 25 CV cycles stability test in DMF with 0.5 M KPF_6 as the supporting electrolyte.

Electrochemistry

Cyclic voltammetry of linkers

Cyclic voltammetry (CV) of free linkers in solution was performed using a one-compartment, three-electrode configuration connected to an Autolab PGSTAT100 potentiostat controlled with Nova 2.1.4 software. The electrode setup includes a glassy carbon disc working electrode, a glassy carbon rod counter electrode, and a non-aqueous Ag/Ag+ reference electrode (10 mM $AgPF_6$ in acetonitrile). Solutions of 0.5M KPF_6 in dry DMF were used as the supporting electrolyte, and argon was bubbled through the solution for 15 min before each experiment.

Cyclic Voltammetry of MOF Thin Film

Electrochemical analyses were performed in a standard three-electrode set-up connected to an Autolab PGSTAT204 potentiostat controlled with Nova 2.1.4 software: the Zn-XDI@FTO (X = PM, N, P) thin films as a working electrode, a glassy carbon as the counter electrode, and a non-aqueous Ag/Ag^+ reference electrode (10 mM $AgPF_6$ in acetonitrile). Solutions of 0.5M KPF_6 in dry DMF were used as the supporting electrolyte. MOF-modified FTO was sonicated for 1 min to remove loosely bound partials prior to any experiments. The electrolytes were directly used as bought and the solvent was taken from the solvent purification system (SPS) without any further purification. Before the experiment, argon was bubbled for 15 min to the electrolyte solution. The headspace of the electrochemical cell was continuously purged with argon during the experiments. The applied potentials were calibrated against the ferrocene $Fc^{+/0}$ redox couple.

Table S1. Summary of formal redox potentials of Zn-XDI@FTO (X = PM, N, P) thin films and linkers in solution.

	Zn-XDI@FTO		XDI in DMF	
	$E_{1/2}^{0/\bullet-}$ (V)	$E_{1/2}^{\bullet-/2-}$ (V)	$E_{1/2}^{0/\bullet-}$ (V)	$E_{1/2}^{\bullet-/2-}$ (V)
PMDI	-1.17	-1.72	-1.16	-1.79
NDI	-0.97	-1.34	-0.94	-1.4
PDI	-0.67	-0.82	-0.65	-0.86

Redox potentials are reported versus $Fc^{+/0}$ and measured in DMF with 0.5 M KPF_6

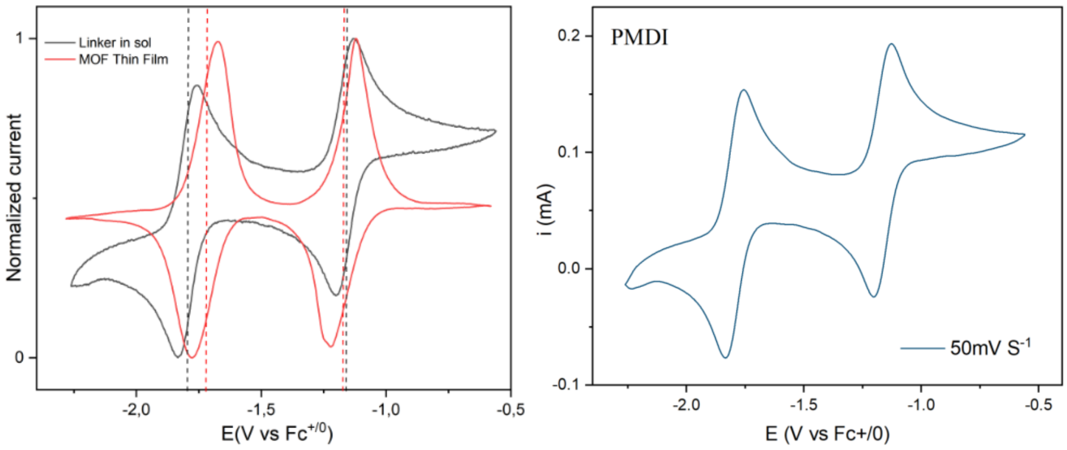

Figure S18. Representative cyclic voltammograms of PMDI linker in solution compared with Zn-PMDI MOF thin film (Left), compared linker in solution only (Right). All measurements were performed in an Ar-saturated DMF solution with 0.5 M KPF_6 as supporting electrolyte, formal redox potentials of the linker and MOF thin film were labeled in black and red dash lines respectively.

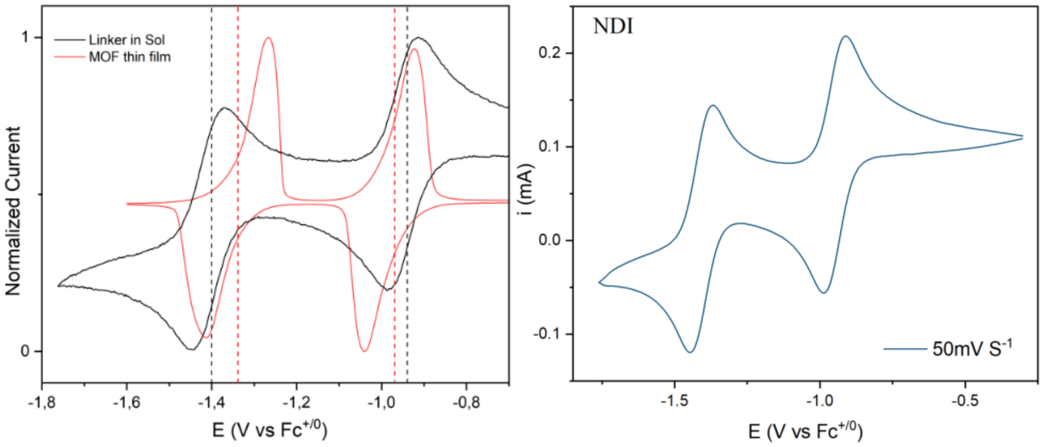

Figure S19. Representative cyclic voltammograms of NDI linker compared with Zn-NDI MOF thin film (Left), compared linker in solution only (Right). All measurements were performed in an Ar-saturated DMF solution with 0.5 M KPF_6 as supporting electrolyte, formal redox potentials of the linker and MOF thin film were labeled in black and red dash lines respectively.

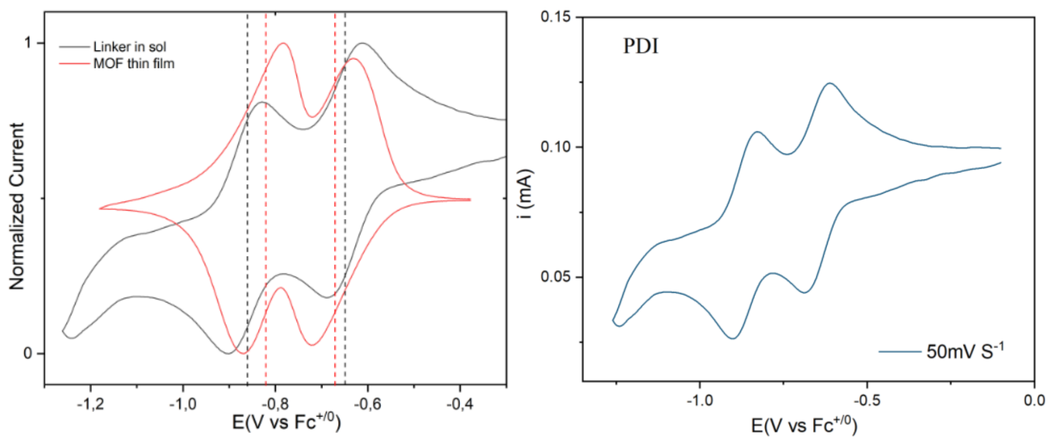

Figure S20. Representative cyclic voltammograms of PDI linker compared with Zn-PDI MOF thin film (Left), compared linker in solution only (Right). All measurements were performed in an Ar-saturated DMF solution with 0.5 M KPF_6 as supporting electrolyte, formal redox potentials of the linker and MOF thin film were labeled in black and red dash lines respectively.

CVs of MOF thin films; transition from finite to semi-infinite diffusion regime

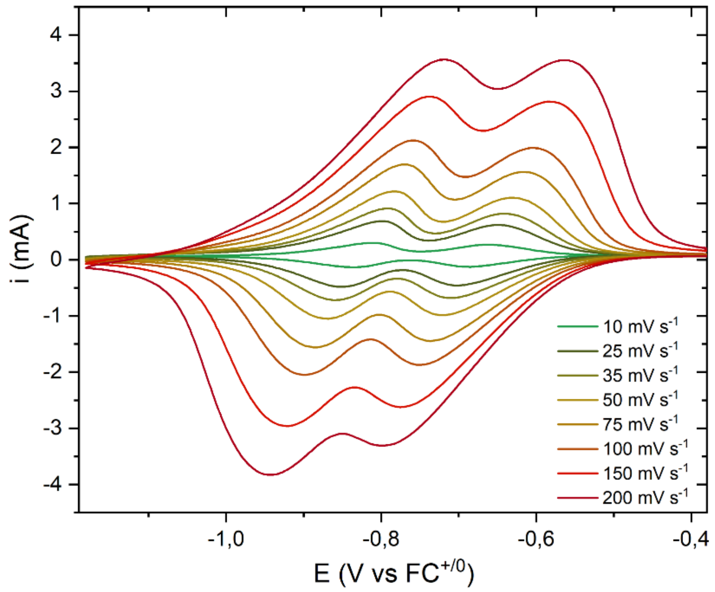

Figure S21. Scan-rate-dependent cyclic voltammogram of Zn-PDI@FTO in Ar-saturated DMF solution with 0.5 M KPF_6 as supporting electrolyte at scan rates from 10 to 200 mV s^{-1}.

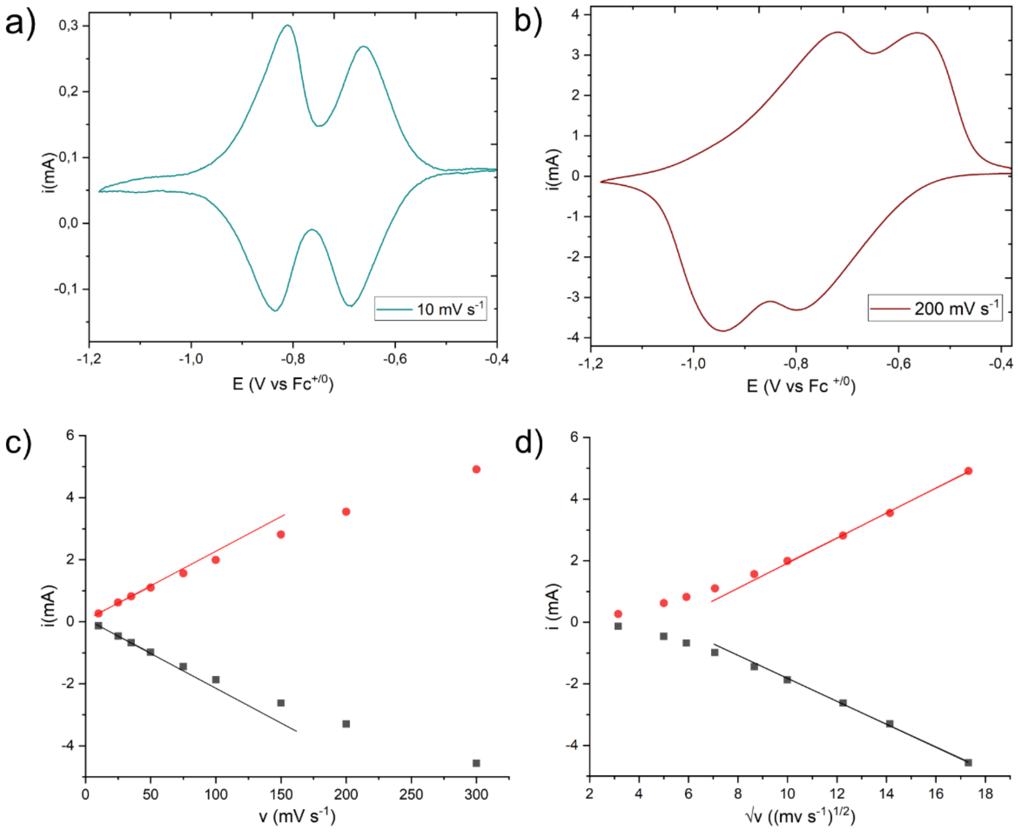

Figure S22. Cyclic voltammograms of Zn-PDI@FTO in Ar-saturated DMF solution with 0.5 M KPF_6 as supporting electrolyte at a scan rate of 10mV s^{-1} (a) and 200mV s^{-1} (b). The plot of cathodic and anodic peak currents corresponding to $[PDI]^{0/\bullet-}$ redox process, *i* **vs.** *v* (c) and *i* **vs.** $v^{-1/2}$ (d), reveals the transition scan rate.

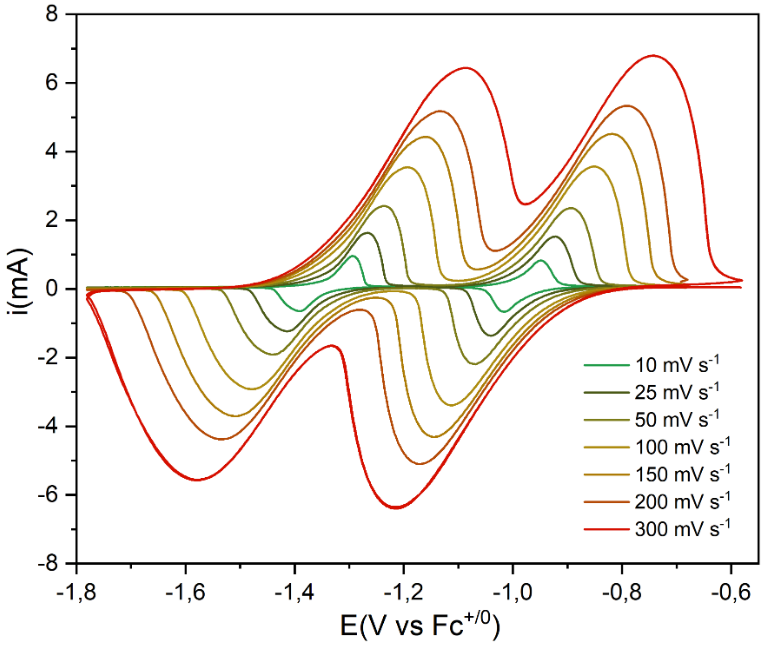

Figure S23. Scan-rate-dependent cyclic voltammograms of Zn-NDI@FTO in Ar-saturated DMF solution with 0.5 M KPF_6 as supporting electrolyte at scan rates from 10 to 300 mV s^{-1}.

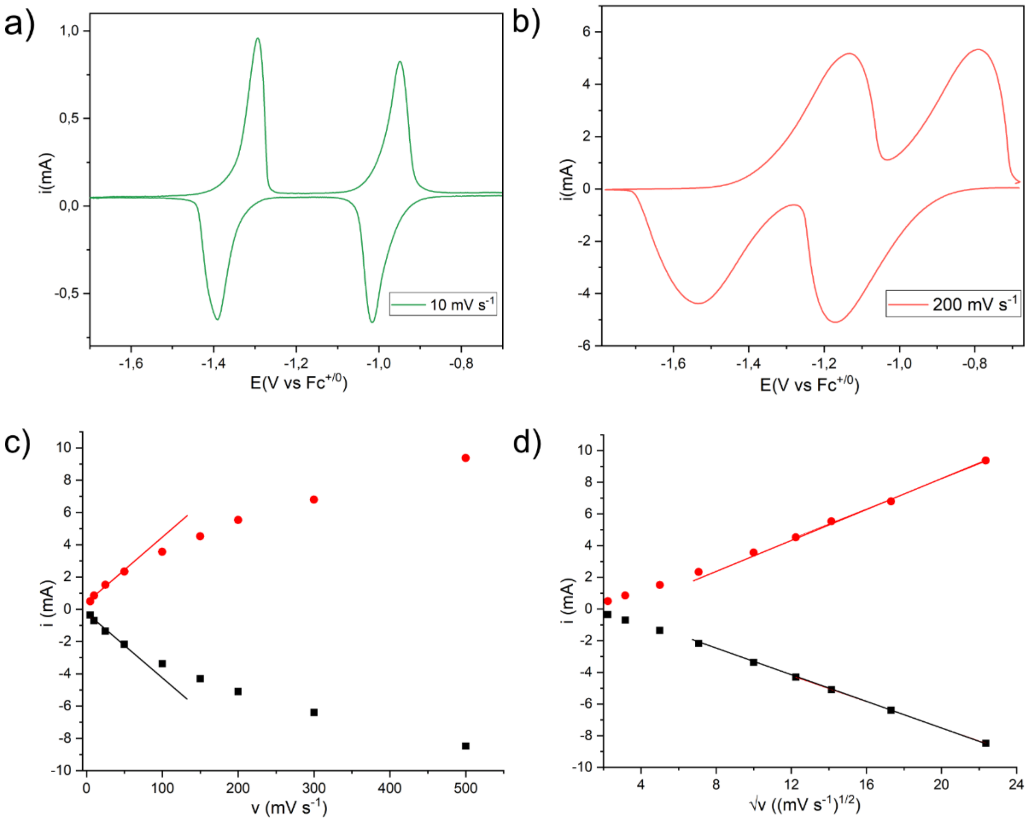

Figure S24. Cyclic voltammograms of Zn-NDI@FTO in Ar-saturated DMF solution with 0.5 M KPF_6 as supporting electrolyte at a scan rate of 10mV s^{-1} (a) and 200mV s^{-1} (b). The plot of cathodic and anodic peak currents corresponding to $[NDI]^{0/\bullet-}$ redox process, *i* **vs.** *v* (c) and *i* **vs.** $v^{-1/2}$ (d), reveals the transition scan rate.

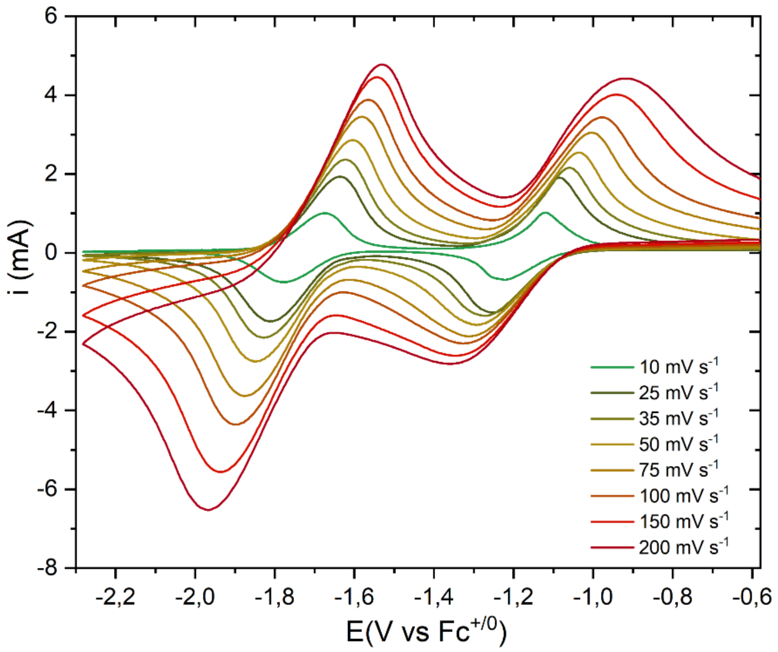

Figure S25. Scan-rate-dependent cyclic voltammograms of Zn-PMDI@FTO in Ar-saturated DMF solution with 0.5 M KPF_6 as supporting electrolyte at scan rates from 10 to 200 mV s^{-1}.

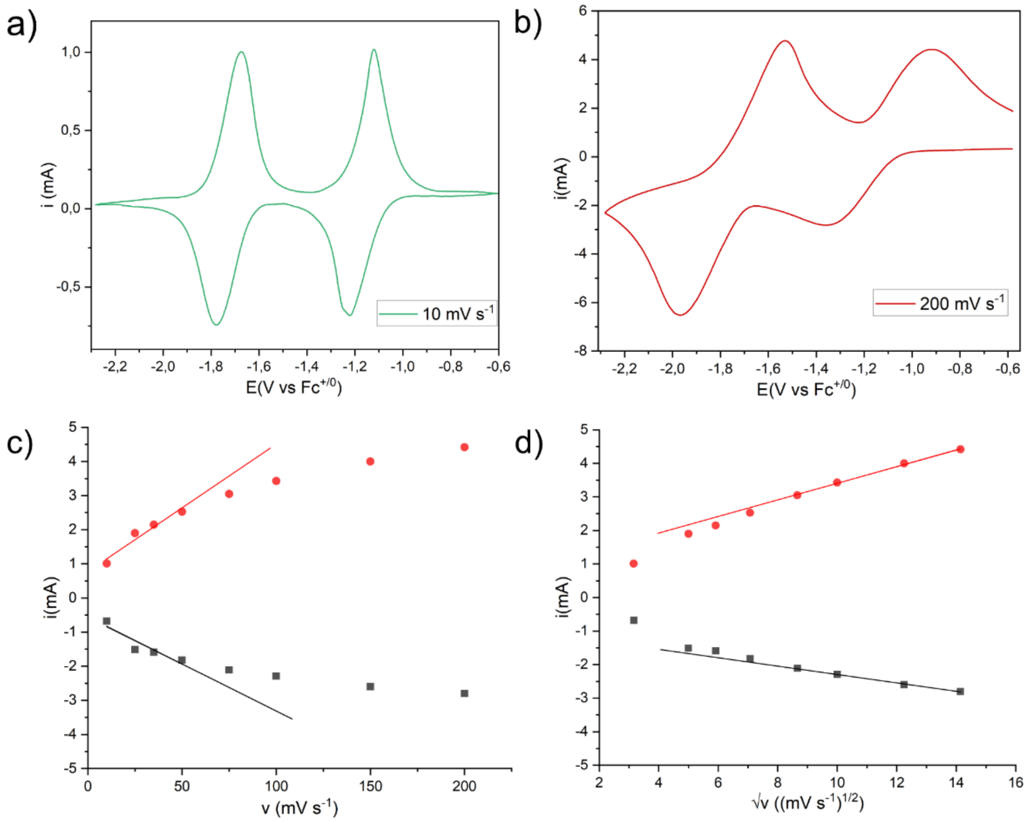

Figure S26. Cyclic voltammograms of Zn-PMDI@FTO in Ar-saturated DMF solution with 0.5 M KPF_6 as supporting electrolyte at a scan rate of 10mV s^{-1} (a) and 200mV s^{-1} (b). The plot of cathodic and anodic peak currents corresponding to $[PDI]^{0/\bullet-}$ redox process, *i* **vs.** *v* (c) and *i* **vs.** $v^{-1/2}$ (d), reveals the transition scan rate.

Spectroelectrochemical analysis (SEC)

Time-resolved UV-Vis spectra of electrogenerated species were collected in-situ using a diode array spectrophotometer (Agilent 8453) coupled to an Autolab PGSTAT100 potentiostat controlled with Nova 2.1.4 software. Redox processes were carried out in a home-made electrochemical cell, consisting of a glass cuvette with 1 cm pathlength and a stopper designed to hold the three electrodes: The Zn-XDI@FTO thin film as working electrode, a Pt rod as the counter electrode, and a non-aqueous Ag/Ag+ reference electrode (10 mM $AgPF_6$ in acetonitrile) (Figure S21), with only the working electrode in the optical path.

The spectroscopic background was collected using bare FTO slides in electrolyte solution under otherwise identical conditions. Before the constant potential step (chronoamperometry) was applied, the sample was allowed to a pre-equilibrate for 50 s at neutral potential. The potentials of the redox processes determined from electrochemical measurements were used as a guide for SEC experiments. To resolve the two redox events and choose an appropriate potential to isolate the $XDI^{0/\bullet-}$ couple for

controlled potential experiments, we measured the CV for each thin film at a very slow scan rate, and a step potential was carefully selected just above the $E_{1/2}$. The chosen step potentials for chronoamperometry are -0.7 V for PDI, which is 30 mV more negative than the redox potential of $PDI^{0/\bullet-}$, -1.1 V for NDI, which is 130 mV more negative than the redox potential of $NDI^{0/\bullet-}$, and -1.35V for PMDI, which is 180 mV more negative than the redox potential of $PMDI^{0/\bullet-}$. These step potentials are carefully selected just beyond the first reductions and the potential is sufficient to reduce all the redox molecule once and don't show any absorption peak for 2nd reduction.

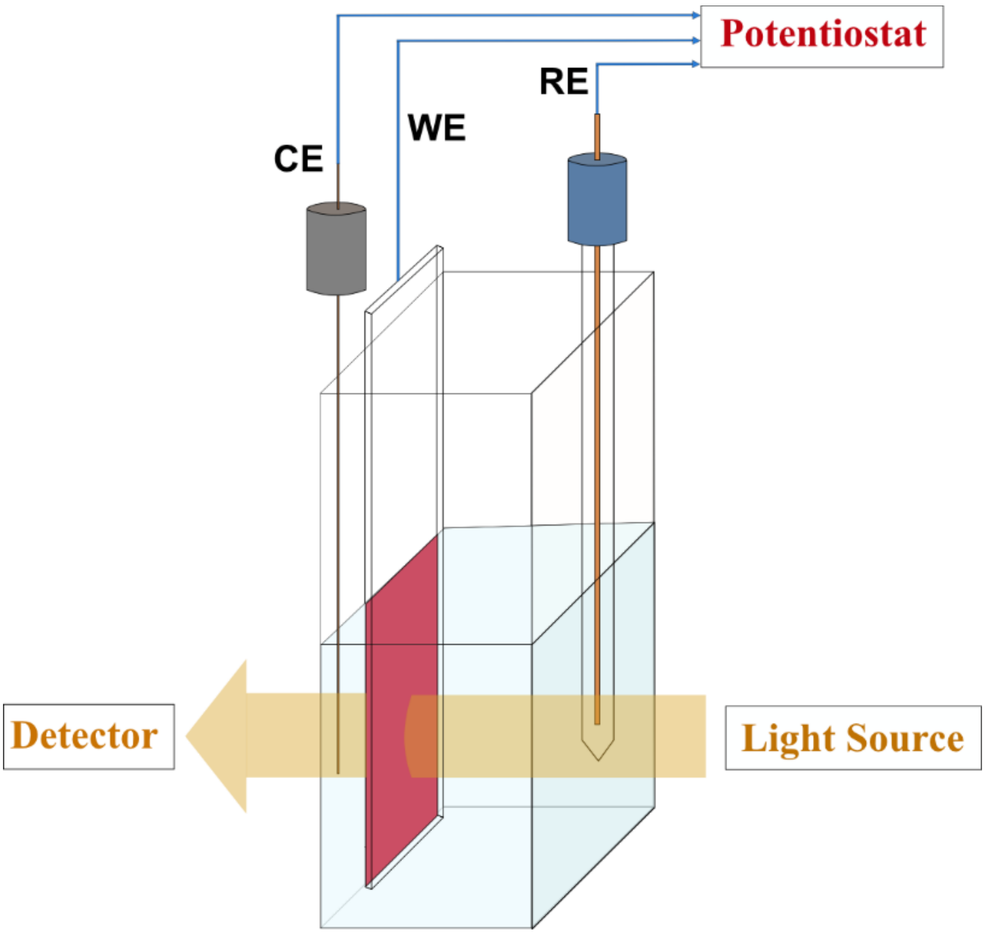

Figure S27. Schematic representation of the 3-electrode quartz cell for the spectroelectrochemical analysis of the Zn-XDI@FTO thin films as working electrode, Pt as counter, and $Ag/AgNO_3$ as reference electrode.

Table S2. Summary of absorption maxima of the free XDI linkers in solution (DMF) and Zn-XDI@FTO (X = PM, N, P) MOF thin films, as determined from spectroelectrochemistry.

		XDI in DMF		Zn-XDI @FTO		
		**Neutral** (λ in nm)		**Neutral** (λ in nm)	**Monoreduced** (λ in nm)	**Bireduced** (λ in nm)
PMDI		270		270	714	549
NDI		359, 379		359, 379	472, 609	396, 418
PDI		485, 519		485, 519	746, 912	667

Apparent diffusion coefficients

A modified version of the Cottrell equation was used to relate the observed change in absorption during spectroelectrochemistry with the apparent diffusion coefficient D_e^{app}, as given in (eq S1). The D_e^{app}s were extracted from the linear regions of the absorption change versus the square root of the time plot (Figure S28). Here, the extracted D_e^{app} values of each Zn-XDI MOF are averages of 3 different film preparations.

$$\Delta A = \frac{2A_{max}}{d_f}\sqrt{\frac{D_e^{app}t}{\pi}} \qquad \text{(Eq. S1)}$$

Here, ΔA is the change in absorbance, A_{max} is the absorbance maximum, t is time in seconds and d_f is the thickness of the film.

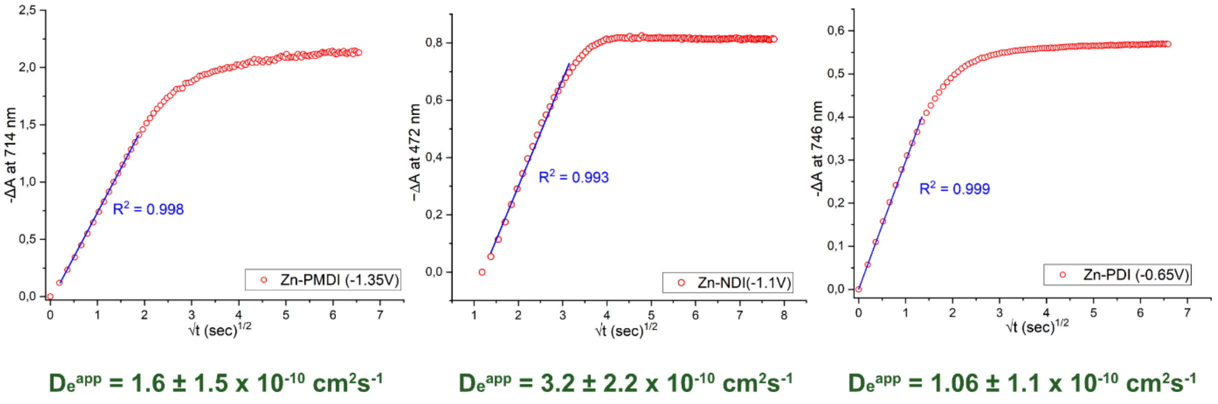

Figure S28: Plots of ΔA vs. $t^{1/2}$ at characteristic wavelength of $[XDI]^{\bullet-}$ for each film. The slope is equal to $2A_{max}\sqrt{D_e^{app}}/d_f\sqrt{\pi}$, which was used to calculate D_e^{app}.

Electrochromic performances

Coloration efficiency and extracted charge

The electrochromic coloration efficiency (η) relates the absorption change to the injected or extracted charge per unit area.

$$\eta = \frac{\Delta OD}{Q} \qquad \text{(Eq. S2)}$$

OD is optical density and Q represents the intercalated/ extracted charge density (C cm^{-2}), here calculated through chronoamperometry by carefully selecting the reduction to avoid extra charge contribution from 2nd reduction.

Coloration efficiency can be extracted by fitting the linear region of ΔOD vs charge density plot. Here and in the manuscript, the calculated error margins are averages of multiple measurements of different thin film samples.

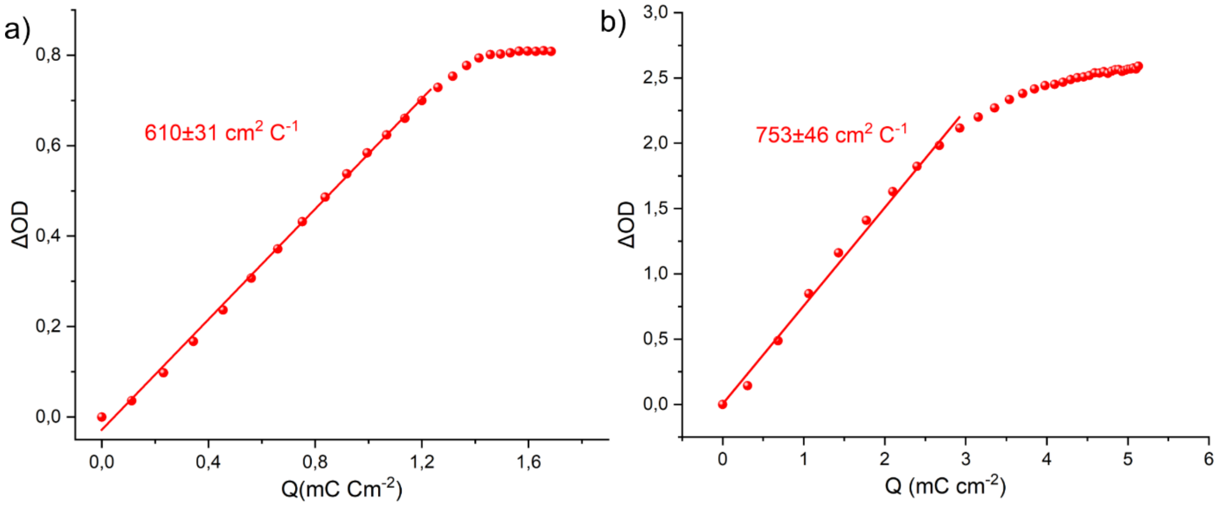

Figure S29: Q is the extracted charge, calculated from the chronoamperometry showing charge passed recorded after stepping potential from a) 0 V to -1.1V for redox couple $NDI^{0/\cdot-}$ b) 0 V to -1.35V for redox couple $PMDI^{0/\cdot-}$

Optical contrast

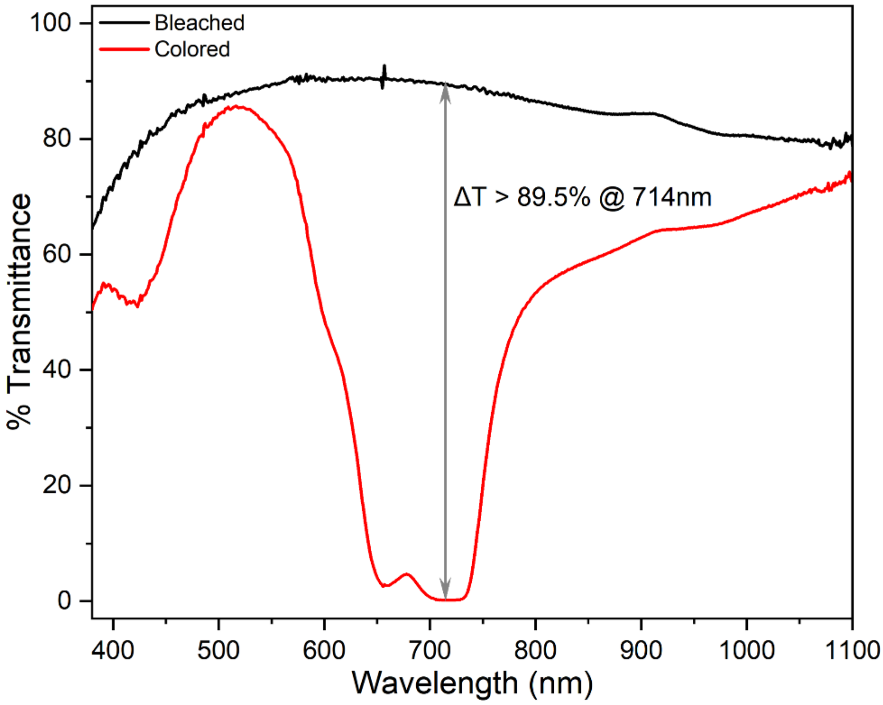

Figure S30: UV−vis transmittance spectra of the Zn-PMDI MOFs film at colored and bleached states.

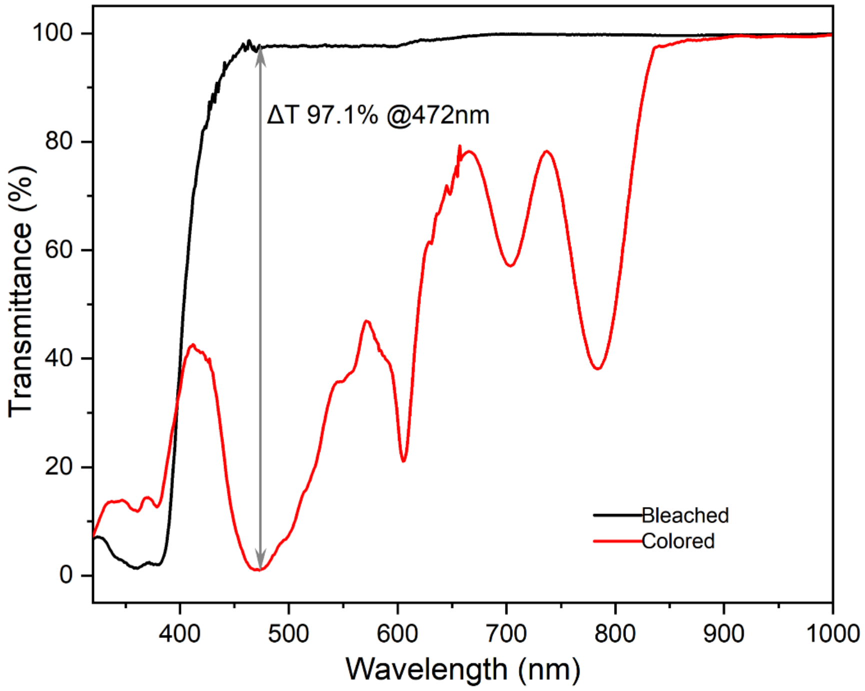

Figure S31: UV−vis transmittance spectra of the Zn-NDI MOFs film at colored and bleached states.

Switching time

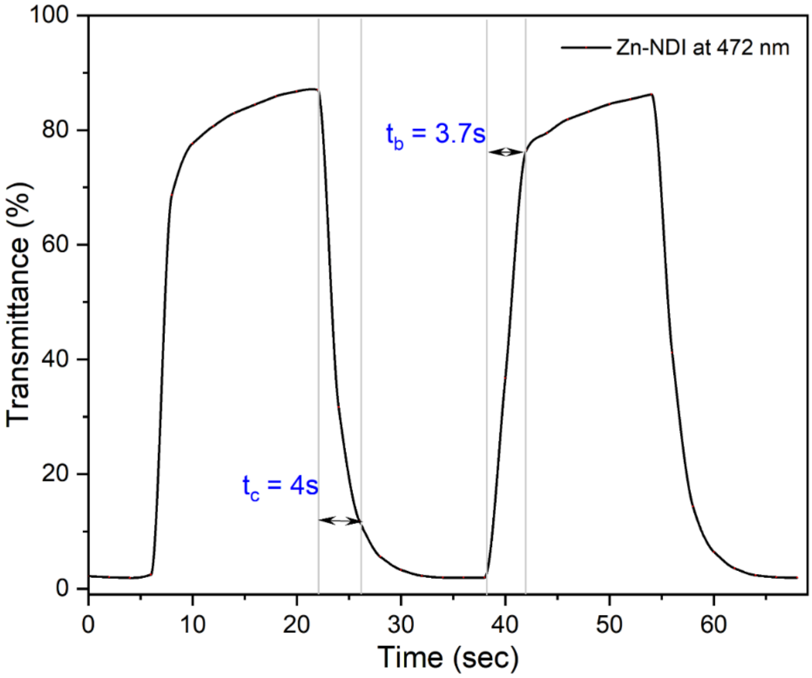

Figure S32. Switching time of Zn-NDI thin film, fast response of 4 s for coloration and 3.7 s for bleaching (monitored at 472 nm).

Spectroelectrochemistry stability test

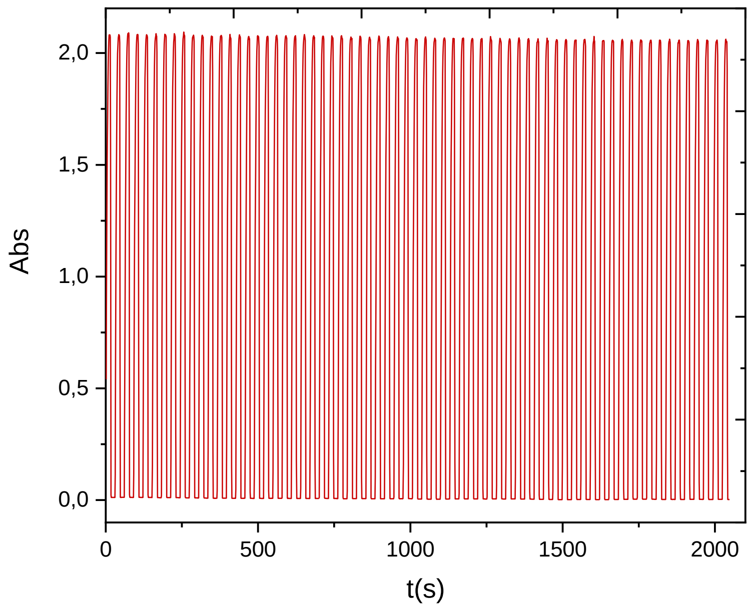

Figure S33. The applied potential is switched between 0V and -0.65V over 100 reduction/ oxidation cycle for Zn-PDI MOF thin film, upon oxidation/reduction the absorbance at 746 nm (characteristic wavelength peak for $[PDI]^{\bullet-}$) only shows a minimal drift (<1%) over 100 cycles.

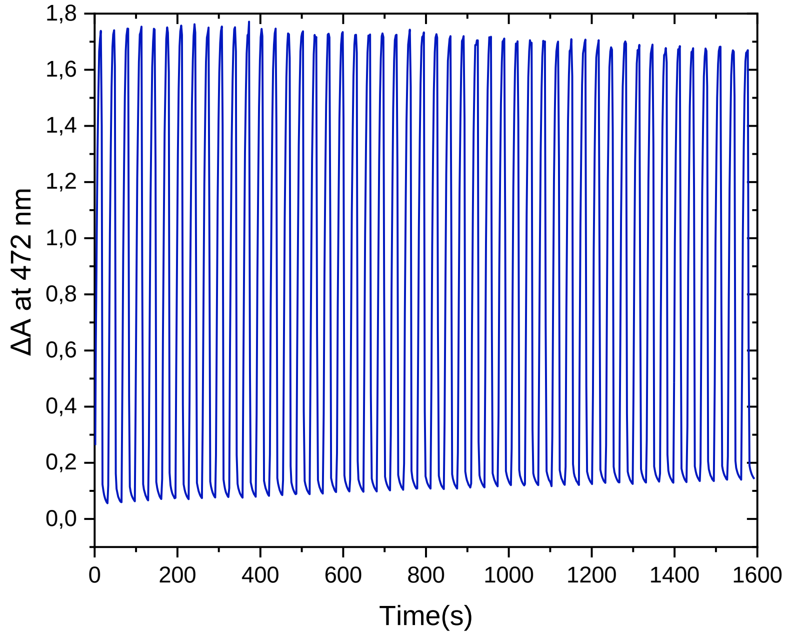

Figure S34. Electrochromic switching stability of Zn-NDI@FTO over 50 oxidation/reduction cycles. The applied potential is switched between 0V and -1.2V. Upon oxidation/reduction, the absorbance at 472 nm (characteristic wavelength peak for $[NDI]^{\bullet-}$) only shows a minimal drift (<4%) over 50 cycles.

Table S3. Summary of MOF-based electrochromic materials in the literature compared to this work.

MOF	t_c/t_b (s)	CE ($cm^2 C^{-1}$)	ΔT_{max} (%)	Cycling number (Retains %)	References
Zn (NDI-H)	~6	297	N.A.	>25	[3]
Zn (NDI-ATZ)	4.35/7.72	99.14	6.45	2000	[4]
Zn-DSNDI (MOF-74 type)	14/23	117	21	>10	[5]
Zn-TCA	4.5/6.2	N.A.	57	140	[6]
Zn-DHTP	10/7	N.A.	31	>100	[7]
Cu-HHTP	3.2/5.9	632	40.2	100(61%)	[8]
Cu-TCA	5	N.A.	65	1000(95%)	[9]
Cu-TTPA	N.A.	N.A.		8	[10]
Cu-BTC(HKAUST-1)/Zn MOF-74	5/6	N.A.	64	>100	[7]
Ni- CHNDI (MOF-74 type)	2.1/1.9	260	73	500(91%)	[11]
NI- BINDI	9.5/5.1	132	42	N.A.	[11]
Ni-HITP	0.6/0.7	530	10	N.A.	[12]
Ni-DHTP	23/29	N.A.	14.6	650(78.5%)	[13]
Ni/Mg-CHNDI (MOF-74 type)	7	>100	N.A.	10	[14]
Mg-PDI (MOF-74 type)	2.4/1.2	200	27.7	1000(79.31)	[15]
Zr- BINDI	N.A.	55.38	29	>5	[16]
Zr-TBAPy (NU-901)	5/12	204	62	60(38%)	[17]
Zn-PDI	1.6/2.6	941	96.4	150(98%)	This Work
Zn-NDI	3.7/4	610	97.1	50(96%)	This Work
Zn-PMDI	N.A.	753	89.5	N.A.	This Work

References

(1) Schneider, C. A.; Rasband, W. S.; Eliceiri, K. W. NIH Image to ImageJ: 25 years of image analysis. *Nat. Methods* **2012**, *9* (7), 671-675.
(2) Wade, C. R.; Corrales-Sanchez, T.; Narayan, T. C.; Dincă, M. Postsynthetic tuning of hydrophilicity in pyrazolate MOFs to modulate water adsorption properties. *Energy Environ. Sci.* **2013**, *6* (7), 2172-2177.
(3) Wade, C. R.; Li, M.; Dinca, M. Facile deposition of multicolored electrochromic metal-organic framework thin films. *Angew. Chem. Int. Ed. Engl.* **2013**, *52* (50), 13377-13381.
(4) More, P. P.; Rathod, P. V.; Puguan, J. M. C.; Kim, H. All-in-one display device with multicolor states derived from NBU-3 MOF/monoalkylated viologen hybrid ionogel material. *Dyes Pigm.* **2021**, *195*, 109730.
(5) Wu, X.; Wang, K.; Lin, J.; Yan, D.; Guo, Z.; Zhan, H. A thin film of naphthalenediimide-based metal-organic framework with electrochromic properties. *J. Colloid Interface Sci.* **2021**, *594*, 73-79.
(6) Liu, J.; Ma, X. Y. D.; Wang, Z.; Xu, L.; Wang, F.; He, C.; Lu, X. Metal–Organic Framework-Based Flexible Devices with Simultaneous Electrochromic and Electrofluorochromic Functions. *ACS Appl. Electron. Mater.* **2021**, *3* (3), 1489-1495.
(7) Mjejri, I.; Doherty, C. M.; Rubio-Martinez, M.; Drisko, G. L.; Rougier, A. Double-Sided Electrochromic Device Based on Metal–Organic Frameworks. *ACS Appl. Mater. Interfaces* **2017**, *9* (46), 39930-39934.
(8) Li, R.; Li, S.; Zhang, Q.; Li, Y.; Wang, H. Layer-by-layer assembled triphenylene-based MOFs films for electrochromic electrode. *Inorg. Chem. Commun.* **2021**, *123*, 108354.
(9) Liu, J.; Daphne Ma, X. Y.; Wang, Z.; Xu, L.; Xu, T.; He, C.; Wang, F.; Lu, X. Highly Stable and Rapid Switching Electrochromic Thin Films Based on Metal–Organic Frameworks with Redox-Active Triphenylamine Ligands. *ACS Appl. Mater. Interfaces* **2020**, *12* (6), 7442-7450.
(10) Ngue, C.-M.; Liu, Y.-H.; Wen, Y.-S.; Leung, M.-K.; Chiu, C.-W.; Lu, K.-L. Spectroelectrochemical studies of the redox active tris[4-(triazol-1-yl)phenyl]amine linker and redox state manipulation of Mn(ii)/Cu(ii) coordination frameworks. *Dalton Trans.* **2019**, *48* (27), 10122-10128.
(11) Li, R.; Li, K.; Wang, G.; Li, L.; Zhang, Q.; Yan, J.; Chen, Y.; Zhang, Q.; Hou, C.; Li, Y.; et al. Ion-Transport Design for High-Performance Na+-Based Electrochromics. *ACS Nano* **2018**, *12* (4), 3759-3768.
(12) Li, M.; Wang, L.; Qi, W.; Liu, Y.; Lin, J. Challenges and Perspectives for Biosensing of Bioaerosol Containing Pathogenic Microorganisms. Micromachines, **2021**; Vol. 12.
(13) Zhang, N.; Jin, Y.; Zhang, Q.; Liu, J.; Zhang, Y.; Wang, H. Direct fabrication of electrochromic Ni-MOF 74 film on ITO with high-stable performance. *Ionics* **2021**, *27* (8), 3655-3662.
(14) AlKaabi, K.; Wade, Casey R.; Dincă, M. Transparent-to-Dark Electrochromic Behavior in Naphthalene-Diimide-Based Mesoporous MOF-74 Analogs. *Chem* **2016**, *1* (2), 264-272.
(15) Lu, Z.; Li, R.; Ping, L.; Bai, Z.; Li, K.; Zhang, Q.; Hou, C.; Li, Y.; Jin, W.; Ling, X.; et al. Ultra-stable ionic-liquid-based electrochromism enabled by metal-organic frameworks. *Cell Reports Phys. Sci.* **2022**, *3* (5).
(16) Radha, G.; Roy, S.; Chakraborty, C.; Aggarwal, H. Electrochromic and photochromic behaviour in a single metal–organic framework containing a redox-active linker. *Chem. Commun.* **2022**, *58* (25), 4024-4027.
(17) Kung, C.-W.; Wang, T. C.; Mondloch, J. E.; Fairen-Jimenez, D.; Gardner, D. M.; Bury, W.; Klingsporn, J. M.; Barnes, J. C.; Van Duyne, R.; Stoddart, J. F.; et al. Metal–Organic Framework Thin Films Composed of Free-Standing Acicular Nanorods Exhibiting Reversible Electrochromism. *Chem. Mater.* **2013**, *25* (24), 5012-5017.
